# Recent Advances in Nanomaterial-Based Self-Healing Electrodes Towards Sensing and Energy Storage Applications

**DOI:** 10.3390/s25072248

**Published:** 2025-04-02

**Authors:** Oresegun Olakunle Ibrahim, Chen Liu, Shulan Zhou, Bo Jin, Zhaotao He, Wenjie Zhao, Qianqian Wang, Sheng Zhang

**Affiliations:** 1 Ningbo Innovation Center, Zhejiang University, Ningbo 315100, China; 12125126@zju.edu.cn (O.O.I.); liuchen@nit.zju.edu.cn (C.L.); 22360469@zju.edu.cn (S.Z.); 22260404@zju.edu.cn (Z.H.); 2 School of Mechanical Engineering, Zhejiang University, Hangzhou 310027, China; bjin@zju.edu.cn (B.J.); 22125146@zju.edu.cn (W.Z.); 3 Faculty of Science and Engineering, University of Nottingham Ningbo China, Ningbo 315100, China; 4 School of Mechanical and Energy Engineering, Ningbo Tech University, Ningbo 315100, China

**Keywords:** self-healing, nanomaterials, sensor, real-time monitoring, electrodes, energy storage

## Abstract

Nanomaterial-based self-healing electrodes have demonstrated significant potential in sensing and energy storage applications due to their ability to withstand electrical breakdowns at high electric fields. However, such electrodes often face mechanical challenges, such as cracking under stress, compromising stability and reliability. This review critically examines nanomaterial-based self-healing mechanisms, focusing on properties and applications in health monitoring, motion sensing, environmental monitoring, and energy storage. By comprehensively reviewing research conducted on dimension-based nanomaterials (OD, 1D, 2D, and 3D) for self-healing electrode applications, this paper aims to provide essential insights into design strategies and performance enhancements afforded by nanoscale dimensions. This review paper highlights the tremendous potential of harnessing dimensional nanomaterials to develop autonomously restoring electrodes for next-generation sensing and energy devices.

## 1. Introduction

Self-healing materials have garnered significant attention in recent years due to their ability to autonomously detect and repair damage, thereby extending the lifespan and reliability of various technologies. These materials mimic biological systems, where self-repair mechanisms are essential for survival and functionality. In the context of electrodes, self-healing capabilities are particularly crucial as they are subjected to mechanical, chemical, and electrochemical stresses during operation, which can lead to crack formation, material degradation, and performance decline [1,2]. Self-healing electrodes have emerged as a transformative solution, offering potential applications in motion monitoring, health tracking, environmental sensing, and energy storage systems [3,4]. By autonomously repairing damage, these electrodes can maintain structural integrity and functionality, addressing the limitations of conventional electrodes that are prone to irreversible degradation over time [5]. Likewise, adding self-healing capabilities to energy storage devices has emerged as a viable tactic to significantly increase the devices’ robustness and functionality, drawing inspiration from the natural healing process [6].

At the core of self-healing materials are chemical and physical principles that enable damage detection and repair. Self-healing mechanisms can be broadly categorized into intrinsic and extrinsic systems. Intrinsic self-healing relies on reversible chemical bonds or supramolecular interactions, such as hydrogen bonding, ionic interactions, or dynamic covalent bonds (e.g., Diels-Alder reactions), which allow the material to heal repeatedly without external intervention [7]. Extrinsic self-healing, on the other hand, involves the incorporation of healing agents, such as microcapsules or vascular networks, which are released upon damage to repair the material. While intrinsic systems offer repeatable healing, they often require specific stimuli (e.g., heat, light, or pH changes) to activate the healing process. Extrinsic systems, though effective, are typically limited to single-use repairs and may not fully restore the material’s original properties [8]. Understanding these fundamental principles is crucial for designing self-healing materials with tailored properties for specific applications.

Despite the promise of self-healing materials, several challenges remain. Current self-healing mechanisms often face limitations in terms of healing efficiency, speed, and scalability. For instance, intrinsic self-healing materials rely on reversible chemical bonds or supramolecular interactions, which may not fully restore mechanical or electrical properties after damage [7]. Extrinsic self-healing systems, which incorporate microcapsules or vascular networks, can repair damage but often require external stimuli or are limited to single-use repairs [8]. These limitations highlight the need for innovative approaches to enhance the performance and practicality of self-healing materials.

The integration of nanomaterials into self-healing electrodes has opened new avenues for overcoming these challenges. Nanomaterials, with their unique size-dependent properties, offer distinct advantages, such as high surface area, enhanced mechanical strength, and tunable chemical and electrochemical properties [9]. These characteristics enable nanomaterials to facilitate self-healing through mechanisms, such as nanoparticle diffusion, reconnection of fractured nanostructures, and surface reconstruction [10,11]. Moreover, the dimensionality of nanomaterials, ranging from zero-dimensional (0D) nanoparticles to one-dimensional (1D) nanowires and two-dimensional (2D) nanosheets, plays a critical role in determining their self-healing behavior. For example, 1D nanomaterials can reconnect at fracture points to restore electrical conductivity, while 2D nanomaterials can undergo surface reconstruction to repair structural damage [12,13]. The dimensional confinement of nanomaterials also accelerates ion diffusion and structural recovery, further enhancing self-healing efficiency [14].

Previous review papers have explored various aspects of self-healing materials and their applications. For instance, Zarepour et al. [15] provided a comprehensive review based on the intriguing characteristics and special powers of self-healing 2D graphene and MXene-based composites. The review carefully examined the latest developments concerning their uses with an emphasis on present issues and potential future developments. Also, Ma et al. [4] conducted a comprehensive review on the applicability and development of self-healing materials. Their review summarized different electrodes, electrolytes, and interfacial layers in recent years, focusing on exploring the feasibility of different self-healing strategies in LBs, discussing the advantages and disadvantages of existing strategies in different parts of batteries. Recently, Liao et al. [16] provided a comprehensive overview of self-healing mechanisms in polymers, discussing fundamental principles and practical applications across diverse fields. More recently, Yao et al. [17] reviewed the role of nanomaterials in self-healing systems, highlighting their potential in flexible electronics and the integration of these materials into commercial technologies.

However, these reviews have not systematically addressed the impact of nanomaterial dimensionality on self-healing mechanisms in electrodes, nor have they provided a detailed comparison of performance across different application areas. Specifically, while existing literature touches upon how the structure and properties of nanomaterials can influence healing efficiencies and electrical performances, a finer granularity concerning the dimensional aspects (one-dimensional, two-dimensional, or three-dimensional structures) has been largely overlooked. This differentiation is crucial, as varying dimensionalities can lead to distinct healing efficiencies, mechanical properties, and functionalities, impacting both sensing and energy storage applications.

Furthermore, the existing reviews frequently emphasize either sensing applications or energy storage independently, often neglecting how advancements in one area can inform the other. Our review seeks to bridge this gap by offering a holistic examination of recent advances in nanomaterial-based self-healing electrodes, focusing on how these innovations serve both the sensing domain and energy storage systems while providing a comparative analysis of their performances across these interconnected applications. By synthesizing these insights, we aim to illuminate the pathways through which nanomaterial dimensionality can be tailored to optimize self-healing responses, thereby enriching the understanding and application of self-healing technologies in flexible electronics and beyond.

The structure of this review is as follows: Section 1 presents a comprehensive discussion of the advances in self-healing mechanisms and their integration into electrode materials employing zero- to three-dimensional nanomaterials. These materials are explored for their applications in health monitoring, motion sensing, environmental monitoring, and energy storage, with a focus on the latest breakthroughs in self-healing electrode technologies for sensing and monitoring systems. Section 2 provides a comparative analysis of the advantages and limitations of nanomaterials across dimensions. Section 3 addresses the critical challenge of balancing mechanical robustness with efficient self-healing kinetics, a key issue for practical applications. Finally, Section 4 examines the current challenges and limitations, providing critical insights into future research directions and concluding with a forward-looking perspective. By systematically addressing the synthesis, integration, and functional performance of dimensional nanomaterials in self-healing electrodes, this review aims to serve as a foundational resource for researchers and engineers advancing this dynamic and interdisciplinary field.

### 1.1. Self-Healing Electrodes Based on 0D Nanomaterials

#### 1.1.1. 0D Nanomaterials for Health Monitoring

Zero-dimensional (0D) nanomaterials, including nanoparticles, quantum dots, and nanodots, have been explored as fillers to impart self-healing properties to electrode materials due to their exceptional characteristics [18]. These self-healing electrodes can repair damage and recover functionality, thereby extending the lifespan of wearable health sensors [19].

Gold nanoparticles (AuNPs) are being extensively studied for their potential to detect biomarkers associated with cancer, neurological diseases, proteins, nucleic acids, and infections. Their excellent ability to conduct electricity makes it easier for electrons to move between where chemical reactions occur and the electrode’s surface. This creates multiple locations where biological sensors can detect small amounts of molecules that indicate cancer [20]. In a study, mussel-inspired catechol chemistry was utilized to create Fe_3_O_4_ magnetic nanodots, which were then combined with polydopamine to create self-healing conductive sheets. The coordination connections between catechol and Fe_3_O_4_ nanodots formed dynamic crosslinks that allowed film fissures to rejoin and cure themselves. With strong adhesive characteristics, the composite sheets could be affixed to the skin as self-healing bioelectrodes [9].

Several self-healing mechanisms using zero-dimensional nanomaterials have been investigated, including hydrogen bonding, ionic interactions, and dynamic covalent bonding [10]. Quantum dots and gold nanoparticles functionalized with organic ligands have been demonstrated to have hydrogen bonding interactions that can reversibly break and re-form following mechanical damage [11]. Transition metal chalcogenide nanocrystals use ionic interactions between charged particles to achieve dynamic self-healing [11]. Meanwhile, dynamic covalent connections produced between organic polymers and zero-dimensional nanomaterials show the ability to perform exchange processes and re-form crosslinks following fracture [12]. Properly selecting the self-healing method is crucial to ensuring optimal electrode performance and healing capabilities [13].

Furthermore, 0D nanomaterials, including carbon quantum dots [14] and molybdenum disulfide nanoparticles [21,22], have been incorporated into polymer composite electrodes using techniques such as spin coating, drop casting, and inkjet printing (Table 1). Studies have shown that incorporating tiny amounts of zero-dimensional nanoparticles into electrodes enhances their ability to conduct electricity, improves how well they can bend without breaking and strengthens their capability to repair themselves [20]. A copolymer electrode integrated with 5% molybdenum disulfide nanoparticles showed failure strain recovery of over 95% after 30 min of room temperature healing [23].

Self-healing electrodes containing 0D nanomaterials have demonstrated potential characteristics for different wearable health monitoring applications, such as heart rate sensors, muscle activity monitors, and skin-based temperature patches [20]. The autonomic self-healing capability enables electrodes to sustain performance even after repeated skin deformation and damage. Furthermore, 0D nanomaterials enable optical transparency for photo-based sensors and enhance biocompatibility for skin interfacing [24]. These new electrode materials that can repair themselves show great potential for creating long-lasting and more reliable health sensors that can be worn as health monitoring devices.

**Figure 1:**
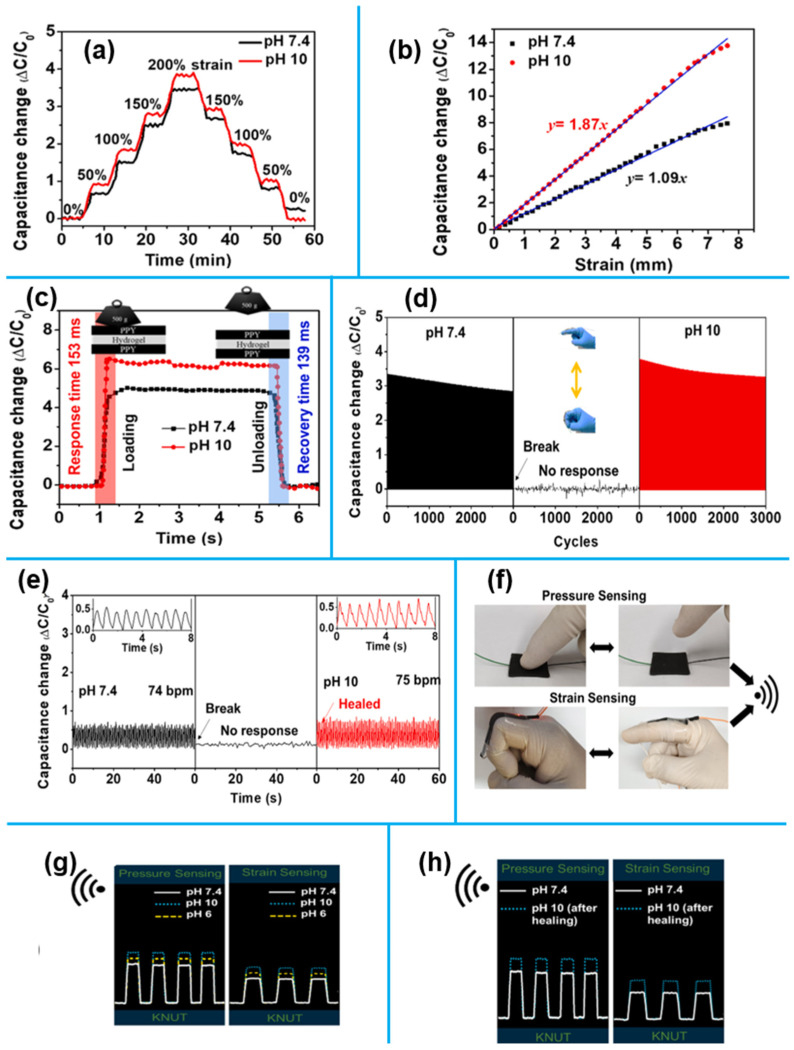
0D Nanomaterial for Health Monitoring (**a**) Changes in relative capacitance over time under varying tensile strain. (**b**) Capacitance variation with strain and its linear fitting curve. (**c**) Response time of the hydrogel capacitive sensor with a 500 g weight applied and released. (**d**) Consistency and repeatability of capacitance response during finger movements. (**e**) Detection of radial pulse using the capacitive sensor. (**f**) Wireless monitoring of pressure and strain during human motion with the hydrogel sensor. (**g**) Wireless monitoring of pressure and strain at different PH levels during finger motion. (**h**) Wireless pressure and strain sensing during finger movements before and after hydrogel healing [25]. Reproduced with permission.

With a near-infrared (NIR)/pH-responsive polymer dot (PD)-embedded hydrogel, a recent study [25] presented a reusable, self-healing, and self-powered electronic skin sensor. This sensor demonstrated high sensitivity and excellent repeatability, with the ability to withstand 12,000 stretch–bend cycles and two cutting-healing cycles. Real-time pH/NIR-dependent sensing was remotely monitored via a smartphone. Integrated with a solar-powered supercapacitor, it achieved 2.32% power conversion efficiency, enabling energy storage during healing. The sensor reliably detected motions ranging from wrist pulses to finger movements, showcasing its potential for wearable health monitoring. Figure 1a depicts how capacitance changes with tensile strains ranging from 0 to 200 percent. As seen in Figure 1b, the hydrogel’s great strain sensitivity was highlighted by the noticeable variations in its relative capacitance that occurred as the strain increased. The pH 10 sensor showed a faster response (153 ms) and recovery periods (139 ms), while the hydrogel sensor made at pH 7.4 showed a longer response (251 ms) and recovery times (273 ms), as shown in Figure 1c. When worn on the wrist, Figure 1e illustrates the real-time sensor curve, and Figure 1f shows the wireless pressure and strain values. Although the hydrogel made at pH 7.4 was more resilient than those made at pH 10 and pH 6, its pressure and strain response was less favorable because of limited particle and ion movement (Figure 1g). These constraints were resolved during the pH/NIR-driven healing process, enhancing the wireless response (Figure 1h).

#### 1.1.2. 0D Nanomaterials for Motion Monitoring

Motion sensors are now widely used in consumer electronics and industrial automation, among other fields of technology [26]. On the other hand, conventional motion sensors deteriorate over time and have a high failure rate. This has sparked tremendous research interest in building self-healing motion sensors that can detect and repair damage automatically [27]. In recent years, zero-dimensional (0D) nanomaterials have emerged as interesting possibilities for allowing self-healing abilities in motion sensors [28].

Quantum dots (QDs) are semiconducting nanocrystals with diameters lower than the exciton Bohr radius, typically 2–10 nm. This quantum confinement phenomenon affords QDs remarkable optoelectronic capabilities, which have been used for self-healing motion sensors [29]. Dai et al. [30] developed flexible motion sensors with near-infrared (NIR) light-emitting CdTe (Cadmium telluride) QDs embedded in polydimethylsiloxane. The QDs functioned as stress-sensitive luminophores, with reversible fluorescence quenching during mechanical deformation. This method allowed the QD-PDMS composites to visibly identify and self-detect damage through variations in NIR emission. Healing was accomplished by allowing the composite to revert to its previous shape, restoring the QD fluorescence.

Incorporating QD microcapsules into polymeric substrates resulted in more advanced self-healing. Furthermore, carbon dots (CDs) are quasi-0D carbon-based nanomaterials that show strong photoluminescence [31]. Their high stability, biocompatibility, and ease of synthesis make them ideal for self-healing motion sensors. In a recent work, CDs were embedded into polyurethane films for visible pressure sensing and damage monitoring [32]. The combination of CD photoluminescence with elastomer matrices provides a simple yet effective approach to self-healing motion sensors. CDs, like QDs, have been encased in microcapsules and placed in self-healing polymer matrices to provide more sophisticated responses. Metallic nanoparticles (NPs) have also been investigated for their ability to provide self-healing and damage-detecting capabilities when put onto motion sensor substrates. Studies on Gold nanoparticles (Au NPs) have been deeply researched over the past years because their localized surface plasmon resonance (LSPR) is highly sensitive to the distance between the particles [33].

Sangeetha et al. [34] deposited Au NP arrays on PDMS membranes to create flexible strain sensors. Tensile deformation induced LSPR shifts and color changes due to nanoparticle rearrangement, enabling visual damage detection. Unloading the strain allowed the NPs to return to their original configuration, healing the damage.

Other metal nanoparticles, such as Ag, have been used for comparable results. For example, ref. [35] created an autonomous self-healing system with Ag NPs in an elastomer matrix. Strain-induced fissures in the material exposed more NPs to air, resulting in the plasmonic oxidation of Ag to Ag_2_O. This chemical change caused a color shift in the sensor, alerting it to damage. Healing was accomplished by using voltage pulses to convert Ag_2_O back to Ag, eliminating the color change.

**Figure 2:**
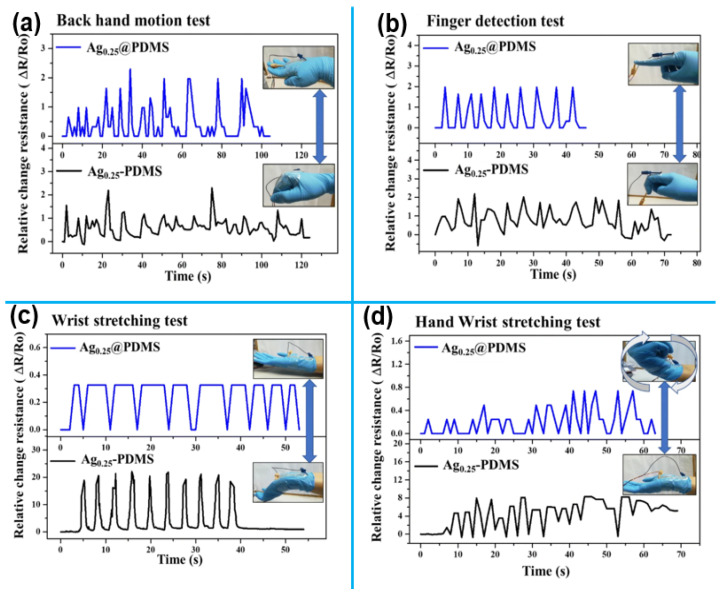
0D Nanomaterials for Motion Monitoring: A comparison of large-strain human motion detection is performed using a strain sensor placed on the finger, hand, and wrist. The images show the relative resistance change during the following tests: (**a**) backhand motion, (**b**) finger insect-like motion, (**c**) forward wrist stretching and (**d**) wrist twisting [36]. Reproduced with permission.

A study by Soe et al. [36] used silver nanoparticles (AgNPs) as conductive fillers on a polydimethylsiloxane (PDMS) substrate to develop resistive strain sensors with a sandwich structure created by the drop-casting method. Two types of sensors were created: PDMS-covered AgNP sensors (Ag-PDMS) and AgNP-modified PDMS composite sensors. The effect of varying AgNP concentrations on PDMS was evaluated, with the Ag0.25@PDMS sensor (0.25 wt% AgNPs) showing optimal performance, achieving a gauge factor of 10.08, 70% strain range, high stretchability, durability, and fast response. Figure 2 compares the sensitivity and response time of Ag-PDMS and Ag@PDMS sensors for real-time human motion detection. Sensors attached to the hand-tracked movements, such as backhand (Figure 2a), finger (Figure 2b), wrist stretching (Figure 2c), and twisting (Figure 2d), with resistance changes analyzed for each motion.

#### 1.1.3. 0D Nanomaterials for Environmental Monitoring

Environmental monitoring is crucial for determining pollution levels and protecting both human and environmental health. Developing strong, reliable sensors is a crucial aspect of environmental monitoring activities [37]. Traditional electrodes in electrochemical sensors decay with time and usage, reducing sensor lifetime and performance. Zero-dimensional (0D) nanomaterial-based self-healing electrodes have been the subject of recent research as a means of extending the stability and lifetime of environmental monitoring sensors [38]. Self-healing electrode designs utilizing 0D nanomaterials have been demonstrated in multiple research as a proof-of-concept. Because of its exceptional electrical properties and surface chemistry, For self-healing applications, graphene quantum dots (GQDs) have been extensively studied [39].

Diverse carbon nanomaterials have been investigated for self-healing electrode designs, apart from graphene. For example, ref. [40] created a self-healing sensor using a carbon nanotube (CNT) sponge electrode. When exposed to mechanical vibration stimuli, the 3D CNT network demonstrated high electrical conductivity and allowed electrode reconnection after damage. This vibration-activated healing enabled the sensor to recover on its own, without the need for external triggers or power. Such strong, autonomous self-healing capabilities may be required for remote environmental monitoring applications where sensor durability and lifetime are crucial.

#### 1.1.4. 0D Nanomaterials for Energy Storage Applications

The high demand for portable electronics and electric vehicles has made energy storage devices essential to modern technology. However, a common challenge is electrode degradation over time, resulting in capacity loss and eventual device failure [41]. Recent research focused on employing zero-dimensional (0D) nanomaterials, such as quantum dots and nanoparticles, as building blocks for self-healing electrodes [42]. Compared to bulk materials, 0D nanomaterials have an unusually large surface area and unique size-dependent characteristics, making them interesting candidates for this application [43]. Quantum dots are extremely tiny crystal structures on the nanoscale, with diameters under 10 nanometers, which makes them smaller than the natural distance between an excited electron and the hole it leaves behind. At this size scale, QDs display distinct optoelectronic characteristics and quantum confinement effects [44]. For self-healing electrodes, the advantage of QDs is their ability to sinter and fuse when subjected to elevated temperatures. This provides a mechanism for autonomously repairing cracks and defects [45].

**Figure 3:**
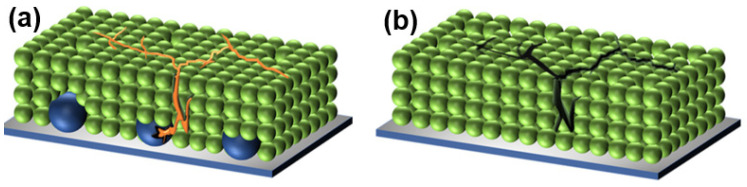
0D Nanomaterials for Energy Storage Devices: (**a**) Picture comparing the pure Si electrode and the GaInSn–MS/Si electrode. (**b**) The electrode after 100 charge-discharge cycles [46]. Reproduced with permission.

Using an aqueous casting technique, ref. [46] developed a self-healing GaInSn–MS/Si hybrid electrode by incorporating microencapsulated gallium, or liquid metal (GaInSn), known for its excellent electrical conductivity and mobility. The LM microcapsules, when combined with Si nanoparticles, enabled a uniform dispersion of GaInSn while maintaining its conductivity and fluidity (Figure 3). The uniformly dispersed LM microcapsules in the GaInSn–MS/Si hybrid electrode minimized expansion and contraction, rebuilt the electron conduction network, and significantly improved the Si electrode’s electrochemical performance. Overall, metal nanoparticles (NPs) have demonstrated the ability to fuse and re-form conductive pathways, enabling self-healing mechanisms in battery and supercapacitor electrodes to mitigate deterioration during prolonged cycling. These unique properties of 0D nanomaterials provide an effective strategy for enhancing the durability of energy storage systems [47].

### 1.2. Self-Healing Electrodes Based on 1D Nanomaterials

#### 1.2.1. 1D Nanomaterials for Health Monitoring

1D nanomaterials, such as carbon nanotubes (CNTs), silver nanowires (AgNWs), copper nanowires (CuNWs), and conducting polymer nanofibers, have been widely investigated for the development of self-healing electrodes due to their unique electrical properties and high aspect ratios, one-dimensional [48,49].

These nanomaterials can provide self-healing capabilities via methods such as reorientation to reconnect broken junctions, cold welding at broken interfaces, and polymer chain reconfiguration across cracks. For example, crossing CNT thin films depends on the remarkable mechanical flexibility of individual CNTs to stretch and reorient across damaged areas [50]. Meanwhile, AgNW and CuNW films utilize cold welding between fractured wire ends to re-establish electrical continuity [51]. Tuning the attributes of 1D nanomaterials, such as their size and surface chemistry, enables control over their self-healing ability.

Several techniques have been used to infuse 1D nanomaterials in self-healing electrode designs. CNTs and nanowires are typically deposited onto substrate surfaces via solution-based processing methods, such as spray coating, spin coating, and dip coating [52]. Annealing the as-deposited nanomaterials improves their electrical conductivity. Advanced fabrication technologies, such as layer-by-layer assembly and inkjet printing, enable precise patterning of 1D nanomaterials into self-healing electrodes [49].

Yao and Zhu [50] developed highly stretchable resistive sensors (ε ≈ 280%) using aligned CNT thin film–PDMS composites. The stretchability resulted from uniform microcrack propagation and the lateral connectivity of CNTs during stretching. They also created ultra-stretchable capacitive sensors (ε ≈ 300%) by combining a percolating CNT network with silicone elastomer composites, with performance attributed to the dielectric layer’s stretchability and the resilience of the CNT-based electrodes. The incorporation of 1D nanomaterial polymer composites significantly enhanced the stretchability of the strain sensors [50,53,54,55]. Self-healing electrodes made of 1D nanomaterials show potential for merging with the skin in health monitoring applications. Flexible CNT and AgNW electrodes were used as self-healing sensors for pulse and respiration monitoring during skin deformation [56]. Additionally, self-healing triboelectric nanogenerators made of CNT-AgNW electrodes have been proposed as self-powered interfaces for monitoring biomechanical signals [48].

**Figure 4:**
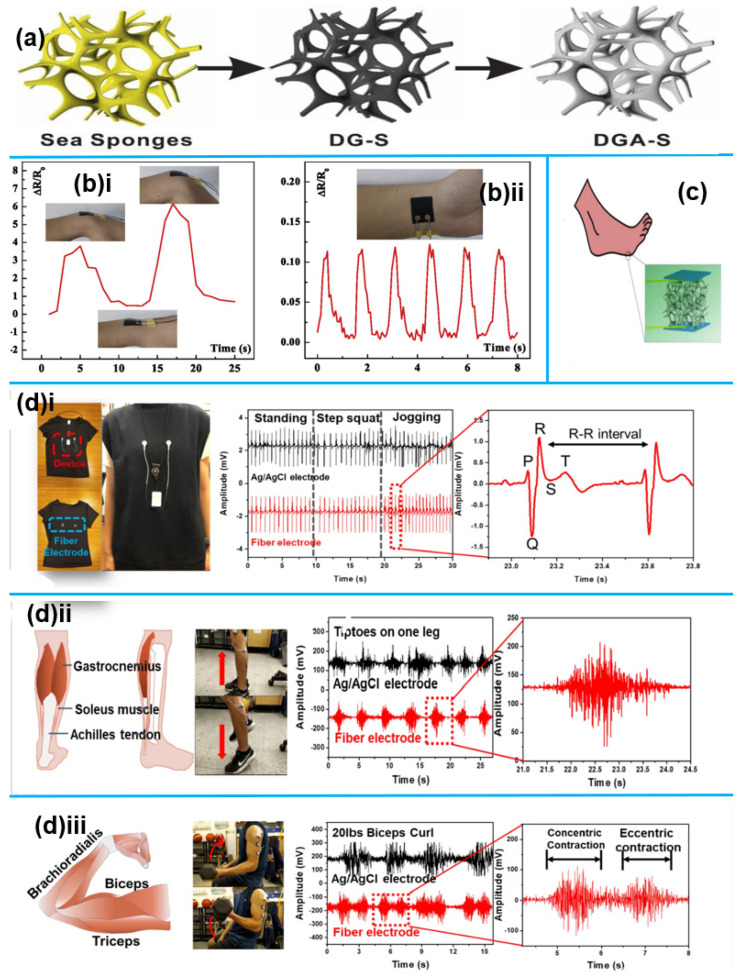
1D Nanomaterials for health monitoring. (**a**) The overall fabrication process of the DGA-S, featuring the dip-coating of pDA-rGO sheets and AgNWs onto sea sponges [59]. Reproduced with permission. (**b**) (**i**). The composite sensor affixed to the wrist for gesture detection; (**b**) (**ii**). The composite sensor is affixed to the wrist for pulse monitoring [57]; (**c**) Schematic diagram of the sensor attached to the foot [59]. Reproduced with permission. (**d**) (**i**). Schematic of physiological signal collection using FHE nanofiber electrodes, attached to the sensor with metal buttons to monitor ECG signals transmitted to a physiological sensing device; (**d**) (**ii**). Calf EMG measurements; (**d**) (**iii**). Electromyographic measures in the arm muscles [60]. Reproduced with permission.

In a similar approach, Huang et al. [57] used CO_2_ laser ablation to produce a pliable, ultra-sensitive piezoresistive strain sensor from a multiwalled carbon nanotube (MWCNT)/polydimethylsiloxane (PDMS) composite. The study thoroughly examined not only the composite’s production via a coating method but also the material’s subsequent electrical and sensing attributes as they were influenced by MWCNT content and laser power. The use of PDMS, a flexible substrate, enabled the creation of a laser-ablated composite sensor for wearable gesture recognition and health monitoring. Figure 4b(i) demonstrates the sensor’s use in wrist gesture recognition. When attached to a volunteer’s wrist (CNT1.0-P3.0), inward bending caused an increase in resistance change (∆R/R_0_), while returning to the neutral position led to a decrease. Figure 4b(ii) illustrates the sensor setup for real-time pulse monitoring.

Furthermore, Gong et al. [58] introduced a cost-effective method for fabricating ultrathin strain sensors using AuNWs, offering excellent stretchability and sensitivity. The production process, compatible with various soft elastomeric substrates, required no specialized equipment or cleanroom environment. The sensor demonstrated a gauge factor (GF) of 9.9, stretchability of 350%, and durability over 5000 cycles. With a broad detection range of 0.01–200%, it effectively monitored strain signals in real time, from subtle skin stretches like radial artery pulses to large muscle movements during various body motions.

In a different study, ref. [59] described a straightforward technique for creating piezoresistive sensors by combining composite conducting networks of silver nanowires (AgNWs) and polydopamine-reduced graphene oxide (pDA-rGO) with hierarchical sea sponge structures. The DGA-S sensor’s total manufacturing process is shown in Figure 4a, where sea sponge skeletons are coated with composite networks of pDA-rGO and 1D AgNWs using a simple dip-coating and drying method. The hierarchical architectures of porous sea sponges, which have multi-scale porous skeletons and bending nodes, established a linear relationship between applied compressive strain and resistance. The sensitivity of this pressure sensor is great (S = 0.016 kPa^−1^ at 0–40 kPa), and its detection range is wide (gauge factor = 1.5 at 0–60% strain). The composite conducting network enhances the sensor’s response time (less than 54 ms) and repeatability (over 7000 loading/unloading cycles). Dong et al. [59] highlighted its potential for health monitoring, demonstrating exceptional stability and flexibility and in tracking both small- and large-scale human movements, (Figure 4c).

In 2023, a study by Li et al. [60] produced AgNP/rGO/PEDOT: PSS/TPU FHE nanofiber electrodes for smart wearable devices to monitor ECG and EMG. As seen in Figure 4d(i), these electrodes were utilized to record EMG signals from the arm and leg. Figure 4d(ii) shows the measurements of the calf muscles. In contrast to Ag/AgCl electrodes, which displayed some noise, the nanofiber electrodes generated distinct EMG signals during muscle action. Figure 4d(iii) shows how the electrodes were also placed on the participant’s arm muscles to track changes in the EMG signal while they lifted a 20-pound dumbbell.

#### 1.2.2. 1D Nanomaterials for Motion Monitoring

Motion sensors play critical roles in robotics, automotive systems, consumer electronics, and industrial monitoring. However, the loss of motion sensor performance over time remains a significant concern. This has sparked interest in self-healing motion sensors, which can detect and fix damage on their own. In addition to their remarkable mechanical strength, carbon nanotubes (CNTs) have great electrical and thermal conductivity and piezoresistive sensitivity [61]. Leveraging these properties, CNTs have been explored extensively for self-healing motion sensors.

Electrospun polymer and carbon nanofibers are very porous, flexible, and stretchable [62]. Piezoresistive nanowires synthesized through controlled growth have long been used to construct self-healing sensors with precision. Au nanowires are significant because of their excellent conductivity and mechanical flexibility [63]. Lee et al. [64] developed vertically aligned Au nanowire arrays on substrates to create transparent and sensitive strain gauges. The nanowire connections were repaired after being fractured under force, restoring sensor conductivity and functionality. Electrical resistance gave immediate input on damage and healing. Epitaxially grown silicon nanowires have also demonstrated similar self-healing capacities as strain sensors [65].

Other studies [66] developed flexible strain sensors with biomimetic polydimethylsiloxane (P-PDMS) patterns and multi-walled carbon nanotubes (MWCNTs) coated with iridium nanoparticles (IrNPs) via atomic layer deposition (ALD). These sensors demonstrated enhanced sensitivity (34.96), a broader sensing range (0–30%), outstanding stability (10,000 cycles), and quick response times (150 ms) following 200 ALD cycles. The enhanced performance resulted from the combined effects of the duck-web-inspired patterned PDMS and Ir@CNTs. To demonstrate its functionality, the sensor was attached to a human thumb, where it detected resistance changes during stretching and bending movements (Figure 4b(i,ii), with consistent resistance variations across different stretch conditions. The sensor can detect both stretching and bending stress. Similarly, when the thumb bends outward, the sensor detects the bending stress and responds quickly and inversely. These findings demonstrate the flexible strain sensor’s ability to quickly and precisely track a range of human actions. Due to their piezoelectric capabilities, semiconducting metal oxide nanowires have also shown potential as self-healing motion sensors. For example, Zhou et al. [67] created flexible strain sensors by embedding SnO_2_ nanowire networks in a polymer matrix. Mechanical deformation caused piezoelectric polarization of the nanowires, which allowed for sensitive motion detection. Partially fractured nanowire networks increased resistance but could be healed by alleviating the strain and allowing nanowire contacts to reconnect.

**Figure 5:**
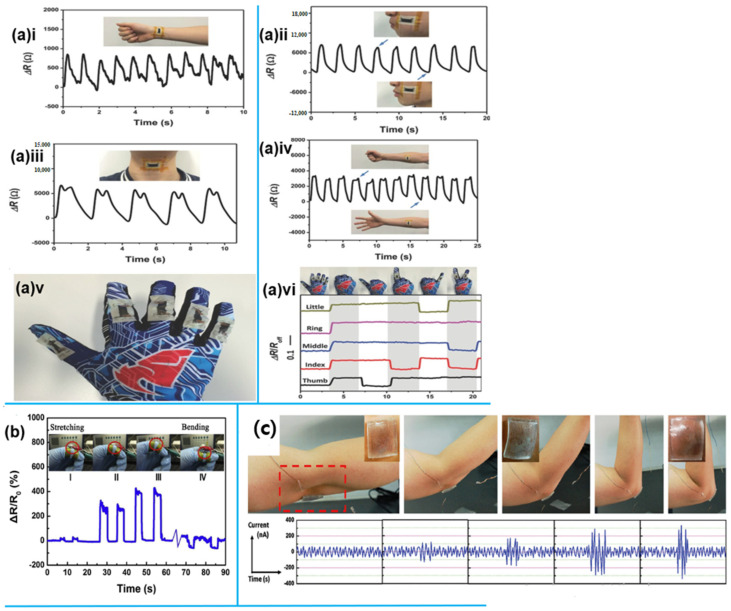
1D Nanomaterials for motion monitoring. (**a**) Resistance fluctuates when the strain sensor is connected to different positions on the human skin. (**i**). Forearm muscle movement; (**ii**). Cheek movement; (**iii**). Throat movement when saying “hello” repeatedly; (**iv**). Human wrist pulse detection; (**v**). Photograph of a glove with strain sensors sewn onto each finger; (**vi**) Resistance changes over time for strain sensors on the glove at six different hand positions [58]. Reproduced with permission. (**b**) Relative resistance change time curve of the 200-cycle IrNPs@MWCNTs strain sensor on patterned PDMS mounted on a thumb under various stretching and bending conditions [66]. Reproduced with permission. (**c**) The strain sensor’s response to detecting human movements, such as the slow movement of the forearm from a straight (horizontal) to a bent (vertical) posture, causing stress at the elbow. The sensor’s response signals demonstrate increasing stress from the forearm movement [68]. Reproduced with permission.

According to a study by [58], strain sensors with a gauge factor (GF) of 6.9–9.9 were produced by using a flexible drop-casting technique to produce 1.64 µm-thin black-gold films on elastomeric sheets like PDMS. With stretchability exceeding 350% and a quick response time of 5000 cycles, these sensors were able to detect strains ranging from 0.01% to 350%. They enabled real-time detection of strain signals, from microscopic skin stretching to large muscle movements. The sensors, easy to attach to the skin, could also be integrated into apparel like gloves for hand motion tracking (Figure 5a). They accurately detected forearm muscle movements (Figure 5a(i)), facial skin stretching during cheek motion (Figure 5a(ii)), and skin stretching on the throat when speaking “Hello” (Figure 5a(iii)). Additionally, the AuNWs strain sensor monitored radial artery pulses in real time, capturing wrist pulses at about 66 beats per minute with high sensitivity and identifiable pulse peaks (Figure 5a(iv)).

G/Ag-NWs hybrid structures were used to create a capacitive strain sensor in a related study [69], and they demonstrated outstanding transparency, flexibility, stretchability, and sensing capability. The sensor uses capacitance changes to detect multidirectional strains from 5% to 200% in less than 1 ms. At stress frequencies between 0.08 and 1.00 Hz, it functions efficiently and responds rapidly with a sharp signal peak. Even under high strains (up to 200%), it retains a steady resistance and uses little power.

When placed on a volunteer’s elbow, the sensor recorded current signals in response to arm movements, corresponding to deformations as the forearm moved between horizontal and vertical positions (Figure 5c).

#### 1.2.3. 1D Nanomaterials for Environmental Monitoring

Environmental monitoring is critical for assessing pollution levels and risks to human and ecological health. Traditionally, metal electrodes have been used for electrochemical sensors in environmental monitoring; however, they can be prone to fouling and degradation over time [69]. Recently, there has been increasing attention on self-healing electrodes made from one-dimensional (1D) nanomaterials as a method to enhance the stability and durability of electrochemical sensors, particularly for long-term environmental monitoring.

For self-healing electrode applications, carbon nanotubes (CNTs) are regarded as extremely promising 1D nanomaterials because of their remarkable conductivity, large surface area, mechanical strength, and flexibility [70]. Studies have shown that CNT conductive networks can self-heal after damage through mechanisms, such as re-stacking and reconnection of displaced CNT bundles [71]. Beyond CNTs, other 1D nanomaterials have also been investigated for self-healing capabilities. Silver nanowires (AgNWs) can form interconnected conductive networks that can re-establish electrical contacts after fracture due to their high aspect ratio morphology [72]. Similarly, tellurium nanostructures, such as nanorods and nanotubes, have shown autonomous self-healing abilities through local melting and reconnecting of broken ends [73]. AgNWs and tellurium nanostructures hold significant potential for creating self-healing electrodes and sensors. For instance, a resistive temperature detector (RTD) was developed using an LCGO/AgNW electrode printed on a PET film sticker. The electrode exhibited a linear resistance–temperature relationship, with a resistance ratio of 1.25 at 100 °C compared to 20 °C. The LCGO/AgNW RTD showcased stability, accuracy, and repeatability due to the metallic properties of AgNWs. Notably, the sensor maintained its performance post-healing, with resistance values consistent with the original at any temperature [74].

In a recent study, AgNW electrodes maintained stable electrochemical sensing of heavy metal ions, such as cadmium and lead, after repeated fracture and self-healing cycles [75]. Such results demonstrate the prospects of self-healing nanomaterial electrodes to improve the stability and extended longevity of electrochemical environmental monitoring sensors. Table 1 below presents an overview of the recent self-healing 0D and 1D used for various application.

**Table 1:** An overview of recent self-healing 0D and 1D Nanomaterials used for health, motion and environmental monitoring.

Materials	Binder	Fabrication	Conductivity S/m/Scm^−1^	Healing Efficiency(%)	Stretchability After Self-Healing (%)	Sensitivity kPa^−1^/mPa	Response Time (ms)	Strain (%)	Gauge Factor	Stability (Cycles)	App.	Ref.
cPDA@ZPD NPs	-	Copolymerization	6.8	2.32	125	0.36	-	585	1.09	-	Health	[25]
MWCNT	PDMS	coating	-	**-**	-	-	-	5.0	513	-	Health	[57]
AgNWs	polydopamine	Self-assembly		-	-	0.016	54	0–60	1.5	-	Health	[59]
FHE nanofiber	PEDOT:PSS	Electrospinning	1.3× 10^1^ Ω/sq	98.3	-	15 mPa	-	200	-	3000	Health	[60]
AgNWs	TPU	Casting	-	-	-	-	-	372	6.78	-	Health	[76]
AgNPs	PDMS	drop-casting	0.11 & 0.14	-	50	2.5		70	10.08	10,000	Motion	[36]
AgNPs	PU	Screen printing	1.65–2.85	-	20	-	-	15–20	-	-	Motion	[77]
AuNWs	elastomeric sheet	Drop-casting	0	-		-	<22	0.01 350	6.9–9.9	5000	Motion	[58]
IrNPs & MWCNTs	P-PDMS	Atomic layer deposition	-	-	-	34.96	150	30	5.12	>10,000	Motion	[66]
G/AgNWs	PAC	bubbletemplate	-	-	200	-	<1	200	22.90	-	Motion	[68]
AgNWs	PDMS	Coating Pre-stretching Drying	-	-	60	-	-	60	150,000	30,000	Motion	[78]
AgNWs	PDMS	Laser cutting Drop coating	-	88.3%		-	-	150	846	1000	Motion	[79]
AgNW/TPU	PDMS	Electrospinning Vacuum filtration Spin coating	50	-	-	-	-	50	12.9	1600	Motion	[80]
AgNWs	TPU	Electrospinning Dip Coating	3990	-	-	-	6	900	44.43	20,000	Motion	[81]
AgNWs	TPU	Spray coating	-	-	-	-	-	100	4.4 × 10^7^	1000	Motion	[82]
AgNWs	PANI/PU	Electrospinning Vacuum filtration	32.09	-	-	-	-	30	59	300	Motion	[83]
AgNWs	Dragon skin	Soft lithography Drop casting	-	-	-	-	-	150	81	10,000	Motion	[84]
LCGO/AgNW	PET	Ink-printing	17,800	95		4.2	-	<1.4	-	-	Environmental	[74]
AgNWs	PDMS	Drop casting	-	-	-	-	-	9	536.98	-	Environmental	[85]

AgNW = silver nanowire; MWCNT = multiwall carbon nanotube; PDMS = polydimethylsiloxane; P-PDMS = biomimetic patterned-polydimethylsiloxane; PET = poly (ethylene terephthalate); FHE = flexible hybrid electronic; cPDA@ZPD = carbonized polydopamine-loaded conductive network containing zwitterionic polymer dot; AgNPs = Silver Nanoparticle.

#### 1.2.4. 1D Nanomaterials for Energy Storage Application

**Figure 6:**
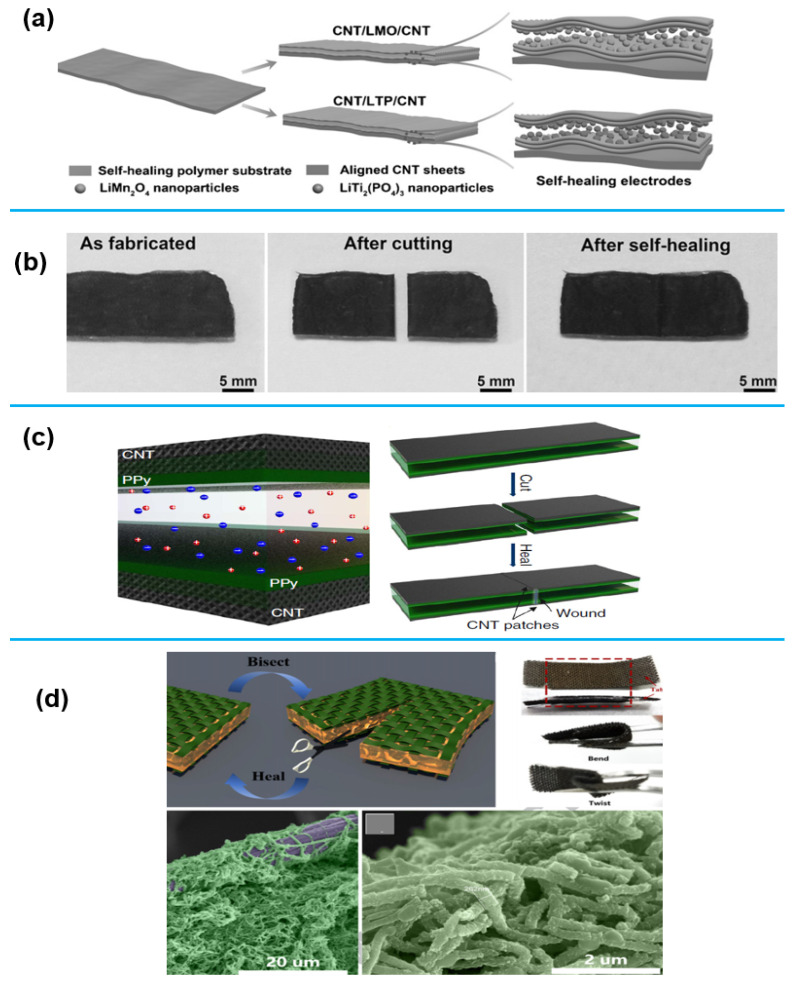
1D Nanomaterials for energy storage application. (**a**) Diagram illustrating the procedures needed to create the electrodes that can mend themselves. (**b**) These electrodes have the remarkable ability to heal themselves both physically and electrically [88]. Reproduced with permission. (**c**) Dual physical crosslinking polyelectrolyte-based self-healing supercapacitor. (**d**) Ferric-enhancing pair of polyelectrolyte-based self-healing supercapacitors [91]. Reproduced with permission.

Two energy storage devices that primarily rely on electrodes are batteries and supercapacitors. However, frequent cycles of charge and discharge can lead to mechanical failure and a loss of electrical contact in the electrodes, reducing their longevity and producing capacity fading [86]. Recently, research has increasingly focused on developing self-healing electrodes using one-dimensional (1D) nanomaterials to enhance the cycle life and stability of these storage devices. 1D nanomaterials, such as nanowires, nanotubes, and nanofibers, have excellent conductivity and mechanical flexibility, which allow them to reconnect damaged circuits through physical touch [87]. The first flexible LIB with self-healing capabilities was presented by Zhao et al. [88]. Figure 6a illustrates the construction of the electrodes, which involved layering CNT sheets onto a self-healing substrate and then incorporating LiMn_2_O_4_ and LiTi_2_(PO_4_)_3_. A gel electrolyte and separator composed of aqueous lithium sulfate/sodium carboxymethylcellulose were utilized in the experiment. This novel battery design showed self-healing properties when it was trimmed six times, causing its capacitance to decline from 28.2 to 17.2 mAh g^−1^ at 0.5 A g^−1^. However, it quickly recovered. In the same vein, it was established in a study by Ezeigwe et al. [13] that one of the biggest challenges of lithium batteries is the measure of their performance degradation of the electrochemical storage which has equally hindered their wide applications. Thus, the development of novel electrodes with self-healing potentials will help to reduce the degradation effect not only in batteries but also in other applications [89,90].

Regarding the electrodes of supercapacitors, ref [91] revealed a polyelectrolyte with dual crosslinking for SC that heals itself. CNT paper coated with polypyrrole (PPy) was employed as an electrode. The constructed SC (Figure 6d) showed strong self-healing properties and capacitance preservation of almost 86% even after the seventh injury. This research demonstrates the potential of carbon nanotubes (CNTs) to produce self-healing supercapacitor electrodes.

Similarly, ref [92] has published work on the dynamic rebuilding of a conductive nanostructure. As soon as the film was removed from the substrate, the conductive CNT network was evident on its upper surface. It was, however, discovered that following the embedding procedure, the CNT network’s resistance increased tenfold, most likely because of some insulating polymer inter-penetration between the CNTs. It was proposed that doping Au salt would increase the electrode’s electrical resistance. To evaluate the self-healing ability of a CNT electrode, the author used in situ monitoring to track damage inflicted by a razor blade. They compared repeated damage (20 times with a 2 N force) and the effects of different forces (0.54 N) applied at various intervals. The results revealed that the damaged CNT electrode could self-repair and restore its functionality after each injury. More crucially, the self-healing polymer matrix demonstrated a broad variety of utility in network restoration, opening the door to longer-lasting electronic applications down the road.

### 1.3. Self-Healing Electrodes Based on 2D Nanomaterials

#### 1.3.1. 2D Nanomaterials for Health Monitoring

Due to its capacity to continuously and non-invasively monitor physiological signals, such as the electrocardiogram (ECG), electromyogram (EMG), and electroencephalogram, wearable health monitoring technologies have become more popular in recent years. Because these wearable devices need to maintain conformal skin contact across bodily motions to acquire high-quality physiological information, flexible and stretchable electrodes are a crucial component [93]. However, the mismatch between the mechanical characteristics of standard rigid electrodes and the soft, curvilinear skin surfaces can lead to motion artifacts and skin discomfort. This has prompted intensive research into creating new stretchy and self-healing electrodes that can sustain good performance even under mechanical deformations from natural motions [94].

Two-dimensional (2D) nanomaterials can create conductive networks (because electrically conductive nanoparticles can be obtained from gold, silver, aluminum, and platinum) that maintain stable electrical properties even under high pressures because of their great electrical conductivity, mechanical flexibility, and nanoscale thickness [95]. Furthermore, recent studies have incorporated dynamic crosslinking chemistries into 2D nanomaterial-based electrodes to achieve self-healing capabilities.

These properties have driven extensive exploration of graphene for stretchable electrodes. Using chemically reduced graphene oxide (CRGO), ref. [96] Created flexible, conductive, dry electrodes for touch-sensing electrocardiograph signal monitoring in a recent study. The proposed method fabricates the device simply and economically by applying CRGO coating to a flexible conducting nylon membrane. The scientists developed the dry electrode sensor to eliminate the inconvenience associated with employing electrolytic gel.

**Figure 7:**
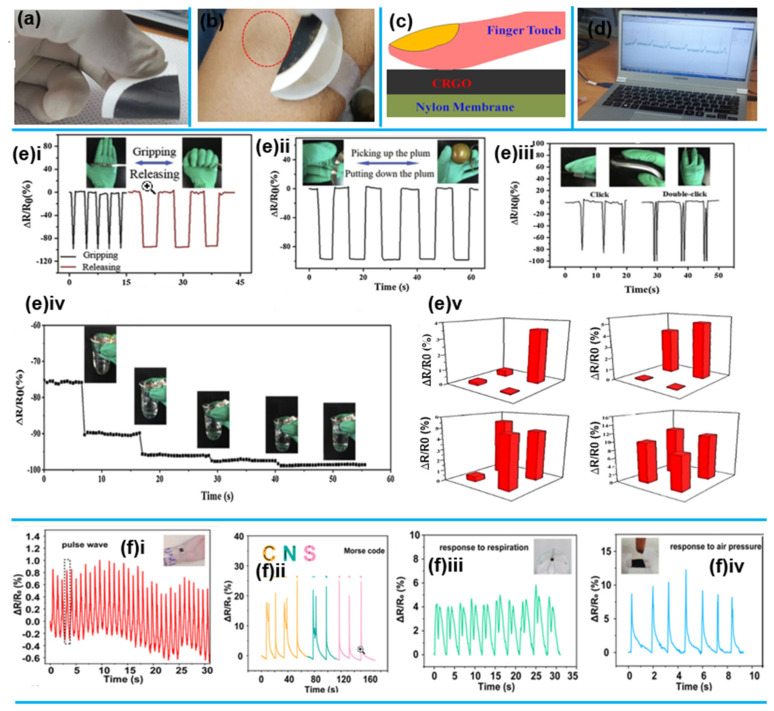
2D Nanomaterials for health monitoring. (**a**) The CRGO electrode’s flexibility. (**b**) Image depicting the skin condition of subjects after three days of continuous use of the CRGO-based dry electrode, and an Illustration of the ECG signal successfully detected from the subject’s finger. (**c**) Touch-sensing mechanism. (**d**) [96] Reproduced with permission. (**e**) (**i**). The CuRGOMF (Curcumin-embedded Real-time Gold Nanosensors for Multifarious Functions) sensor’s response signals throughout cycles of gripping and releasing. (**ii**). The CuRGOMF sensor’s reaction signals when lifting and lowering the plum cycles. (**iii**)**.** The CuRGOMF sensor’s reaction signals upon mouse clicks. (**iv**). The CuRGOMF sensor’s response in identifying pressure distribution (**v**). A CuRGOMF sensor’s response when the beaker is filled with different weights of water [101]. Reproduced with permission. (**f**) (**i**)**.** Wearable real-time wrist pulse monitoring. (**ii**). GSCA knocking to decipher the Morse code. The black dots and curves incorporated into the added letters ingeniously spell out “C N S” in Morse code. (**iii**). The experiment explores the interaction between a participant’s breath and a commercially available mask, focusing on the pressure response generated by a rubber suction bulb [100]. Reproduced with permission.

Graphene, a single sheet of sp2-bonded carbon atoms, has an amazing tensile strength (1TPa), remarkable electrical conductivity (104 S/cm), and intrinsic flexibility due to its distinctive honeycomb structure. These remarkable properties make graphene a promising material for use in flexible electronics, energy storage devices, and self-healing electrodes [97]. To ensure high-quality and reliable physiological measurements, it is imperative to minimize the skin-electrode contact impedance. Figure 7b illustrates the employment of human fingers to maintain good contact in place of CRGO electrodes as touch sensors, as shown in Figure 7a. The CRGO electrode was discovered to have a surface resistance of 28 Ω/square, which is wearable and sufficiently flexible to be utilized for prolonged ECG readings, per the test results. Figure 7c provides a schematic representation of the touch-sensing system incorporating CRGO electrodes. The ECG signal, captured on the monitor during subject interaction with the CRGO electrode, is presented in Figure 7d. High-quality biopotential ECG signal recording is possible using CRGO electrodes without the requirement for skin washing. It can also be applied to EOG, EMG, and EEG data.

MoS_2_ was placed onto pre-strained elastomer substrates by Son et al. [98] to create wavy, buckled morphologies. The MoS_2_ conformally bonded to the elastomer surface upon release. Stretchability up to 50% was made possible by the wavy shape, significantly more than the intrinsic ~1% tensile strain limit of flat MoS_2_ sheets. Chemical doping is an additional way to improve 2D nanomaterials’ electrical conductivity. To accomplish self-healing, recent research has integrated dynamic crosslinking chemicals into 2D nanomaterial-based electrodes.

In a groundbreaking study, Verma et al. [99] presented a novel method for creating wearable strain sensors exploring silicone sealant as a substrate and rGO as a conducting layer. These rGO sealants possess exceptional stretchability (120%), remarkable durability (over 1600 cycles), and high sensitivity (gauge factor > 4000), making them highly promising for various wearable sensor applications. Their superiority in accurately identifying bodily actions under high and low strain stems from this. Bending, stretching, and touching responses were quite stable in the manufactured sensors. The sensors have an extremely fast response speed of 395 milliseconds. They can detect subtle movements such as bending fingers, twisting wrists, folding arms, stretching knees, and bending ankles, without any noticeable delay. The said sealant sensor also proved to be effective in detecting heartbeats and finger touches.

In a significant advancement, ref [100] fabricated a flexible graphene sensor with a GSCA for health monitoring using the DIW technique. Figure 7f(i) demonstrates the GSCA worn on a female’s wrist, enabling real-time monitoring of pulse beats, and showcasing its potential for practical applications. The data indicate a pulse rate of approximately 68 beats per minute. Therefore, the printed GSCA could be utilized for medical monitoring, particularly for individuals with heart disease, asthma, or other cardiovascular conditions. Figure 7f(ii) shows how to decode the “CNS” Morse code. The sensor can be placed on surfaces to collect the necessary private data and can be utilized as a medium for deciphering Morse code. When pressure is applied instantaneously, the GSCA’s output shows one response peak; when pressure is applied gradually, it shows two response peaks. In Figure 7f(iii), a “two peaks” response is observed from a GSCA integrated into a commercial mask, which corresponds to the typical pattern of human respiration. Visible changes in signals can be observed as a result of the GSCA deforming due to the pressure generated by expiratory air. As a result, the sensor may be utilized to track the frequency and intensity of breathing to diagnose patients at home or at the hospital. Figure 7f(iv) illustrates how well the sensor responds to air pressure caused by airflow.

The study [101] introduced a method for making flexible pressure sensors by growing copper nanowires (Cu NWs) in melamine foam (MF) and coating them with reduced graphene oxide (RGO), resulting in CuRGOMF. This sensor showed high sensitivity, detecting pressures from 0 to 18 kPa with varying sensitivities across pressure ranges. It also exhibited outstanding cyclic stability, maintaining its performance over 5000 cycles. To evaluate the CuRGOMF sensor’s response to pressure variations, it was positioned on the palm. The sensor’s rapid response time is demonstrated in Figure 7e(i), where a peak-shaped signal was generated when the palm was quickly gripped. The sensor was then placed on the index finger, and Figure 7e(ii) shows that its resistance decreased when a plum was lifted and returned to its original level once the plum was set down. In Figure 7e(iii), when the sensor was placed on the finger and the mouse was clicked, the sensor’s resistance decreased. The sensor also exhibited a more significant decrease in resistance when the finger clicked twice, confirming that it can accurately detect the pressure from the clicks. Figure 7e(iv) showcases the pixelation of CuRGOMF sensors into a 2 × 2 array to capture spatial pressure, demonstrating the sensor’s capability for space pressure distribution sensing. The sensor array was subjected to various weights to assess its ability to detect spatial pressure distribution. The sensor successfully identified the pressure distribution of different objects based on their height. Figure 7e(v) shows the sensor attached to a forefinger; the thumb and forefinger were used to grip the beakers, each holding a different volume of water. The additional power to hold on is caused by the change in the elastomer’s resistance brought on by the beaker’s increased water volume.

The study [97] described an in-plane integrated dual-mode sensor and a pressure sensor with a vertically integrated electrocardiogram (ECG) electrode made up of a fully self-healing sensor patch. By carefully choosing the sensor design and related functional materials, this work shows how to easily fabricate a high-performance, completely self-healing patch of many sensors, verifying its great potential for use with extremely robust health monitoring systems. For complete self-healing of the device, self-healing oxime-carbamate bond-based polyurethane and room temperature self-healing TUEG3 capped Au nanosheet (T-Au NS) electrodes were used to create interlayer self-bonding. A self-healing dual-mode sensor was also created to detect temperature and pressure concurrently without interference by applying a self-healing composite of polyurethane and polyaniline to a microporous graphene foam by the use of a self-healing sensor foam. Additionally, the pressure sensor was equipped with the interdigitated self-healing electrode of T-Au NS to increase sensitivity up to 208.62 kPa^−1^ (<1 kPa), allowing for the detection of tiny pulse signals even after many self-healing events. ECG signals were also picked up by employing the widely used self-healing T-Au NS electrode, which is vertically integrated into the sensor patch. Following a simultaneous full-device self-healing from full bisection, this sensor patch successfully detects skin temperature, wrist pulse, and ECG signals.

Most studies have only demonstrated self-healing of cuts and scratches. Achieving self-healing after more severe mechanical damage, such as electrode delamination, remains an open challenge. The effects of self-healing on long-term performance and biocompatibility for on-skin use require further investigation. Scalable and low-cost manufacturing methods must be developed to enable commercialization.

#### 1.3.2. 2D Nanomaterials for Motion Monitoring

**Figure 8:**
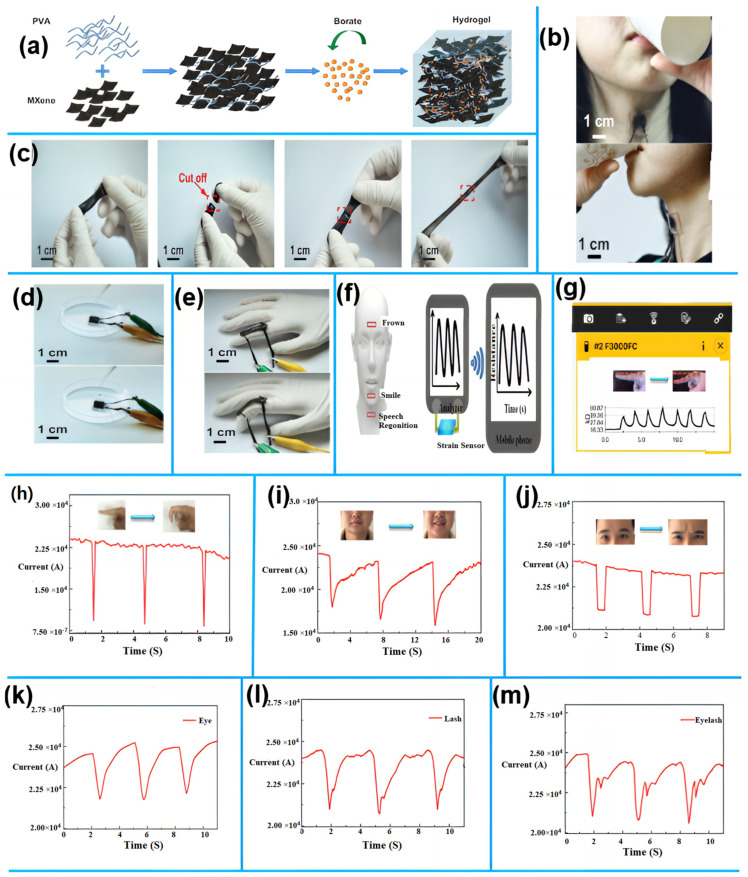
2D Nanomaterial for motion monitoring. (**a**) A diagram illustrating the MXene/PVA hydrogel’s synthesis. (**b**) pictures showcasing the hydrogels’ ability to stretch and self-heal, respectively. (**c**) Pictures of the sensor that tracks the epidermal movement brought on by drinking. (**d**) Images of the water droplet descent sensor. (**e**) Pictures of a motion test using a MXene/PVA capacitive sensor attached to a finger [102]. Reproduced with permission. (**f**) Diagrams showing the strain sensor’s test site. (**g**) Sending sensor signals to the mobile phone via wireless connection for motion detection. (**h**) The sensor’s real-time fluctuations in relative resistance in response to finger bending. (**i**) Present reactions of the initial strain sensors used to detect motion in human speech recognition [111]. Reproduced with permission. (**j**) Facial emotion recognition at various face areas (**k**–**m**).

A noteworthy development was made by [102], who presented a highly adaptable and self-healing MXene/polyvinyl alcohol (PVA) hydrogel electrode especially made for capacitive strain sensors in E-skin applications (Figure 8a). This MXene/PVA hydrogel electrode, as seen in Figure 8b, has exceptional stretchability of about 1200% and can quickly self-heal in just 0.15 s. With the help of these electrodes, capacitive sensors with a sensitivity of about 0.40, low hysteresis, great linearity (up to 200%), and remarkable mechanical durability (only a 5.8% decrease in relative capacitance change after 10,000 cycles) may be developed. The sensor demonstrates its potential for human motion monitoring applications by passing a self-healing test with success and continuing to operate. The device was worn around the neck of a doctoral student to monitor the movement of her throat knot as she drank (Figure 8c). Figure 8d illustrates how relative capacitance fluctuation can be determined when a water droplet falls from a height of 15 cm. One large-strain motion that was detected by the capacitive sensor is finger bending (Figure 8e). An attached capacitive gadget on a rubber glove senses the bending action of the fingers.

An electrode made of MXene/polyvinyl alcohol (PVA) hydrogel that is highly flexible and self-healing was created recently for use in capacitive strain sensors in electronic skin (E-skin) applications. Achieving 1200% stretchability and healing in just 0.15 s, the hydrogel’s conductivity and self-healing qualities were enhanced by the MXene insertion. With a sensitivity of 0.40 and low hysteresis, the resulting capacitive sensor demonstrated great linearity (200%). After 10,000 cycles, the sensor still had 94.2% of its original capacitance, indicating exceptional mechanical endurance. It is interesting to note that the sensor maintains its functionality during a self-healing test, indicating its potential for recording human mobility in real time. For instance, it was utilized to track movements of the throat while drinking (Figure 8c) and was able to detect changes in capacitance when a droplet of water fell from 15 cm (Figure 8d). Furthermore, when connected to a rubber glove, the sensor recorded large-strain movements, such as finger bending (Figure 8e). These findings demonstrate the potential uses of self-healing hydrogels based on MXene in sensitive, long-lasting, and flexible electronic skin for tracking human movement.

Graphene’s exceptional electrical conductivity, strength, and flexibility have made it a perfect nanomaterial for self-healing motion sensors [103]. Cao and colleagues [104] created a graphene Kirigami strain sensor that demonstrated complete mechanical and electrical property recovery following damage.

Furthermore, the sensor also tracked subtle vocal cord movements during pronunciation, showing distinct current patterns for different words (e.g., “eye”, “lash”, “eyelash”) (Figure 8h–k). Even after 1000 stretching cycles or self-healing, the sensor maintained its sensitivity and electrical performance, detecting the same human motions. When integrated onto the volunteer’s forefinger and wrist, it effectively monitored movements like finger bending (Figure 8d), demonstrating its suitability for tracking joint motions. This study underscores the versatility and durability of self-healing GN-based strain sensors for applications, such as facial expression monitoring, vocal cord detection, and joint motion sensing.

Additionally, the transition metal dichalcogenide MoS_2_ has gained attention for its piezoelectric properties useful for self-healing motion sensors [105]. The exploration of a few layers of black phosphorus (BP) is another promising 2D material for self-healing sensors due to its in-plane anisotropic electrical properties highly sensitive to strain [106]. Liu et al. [107] developed a similar BP sensor that could heal after repeated fractures. Hybrid structures of BP with graphene and h-BN have also been explored to improve the self-healing capabilities. Kirigami patterning improved stretchability, and graphene allowed for self-healing by reuniting fractured surfaces. Graphene has also been combined with shape–memory polymers and liquid metals to create dynamic self-healing sensor systems [106].

In a similar study, ref. [107] developed a graphene oxide-nanostructured network (GN) embedded in a rubber matrix, using metal-ligand coordination to enhance its proper-ties. The resulting nanocomposites were five times stronger than the control sample and displayed excellent self-healing capabilities, even under extreme conditions, such as underwater, subzero temperatures, and exposure to acidic or alkaline environments. The strain sensor showed exceptional sensitivity with a gauge factor of about 45,573.1 and was used to monitor facial expressions, such as smiling (Figure 8g) and frowning (Figure 8h), maintaining consistent and repeatable current patterns.

Over the years, 2D insulator h-BN has gained interest for self-healing motion sensors, as its ionic conductivity facilitates hydrogel-like healing. For example, Wang et al. [108] synthesized ionically conductive h-BN for self-healable flexible strain sensors. Dynamic ion migration healed fractures in h-BN, restoring electrical conductivity and strain sensitivity. Fibrous h-BN networks exhibit enhanced damage resilience and healing [109]. Further, h-BN healing has been integrated with graphene piezoresistivty and liquid metal conductivity for sophisticated sensor self-healing [110].

#### 1.3.3. 2D Nanomaterials for Environmental Monitoring

Environmental monitoring is critical for assessing pollution levels and preserving ecological and human health. Electrochemical sensors play a key role in environmental analysis due to their high sensitivity, low fabrication cost, and portability [112]. However, electrodes’ long-term stability and repeatability are often limited by fouling, degradation, and dynamic environmental conditions [113]. Several studies have focused on developing self-healing electrodes using two-dimensional (2D) nanomaterials to improve robustness. Graphene is a 2D carbon nanomaterial renowned for its high conductivity, flexibility, and mechanical strength. These properties make graphene well suited for self-healing electrode designs.

**Figure 9:**
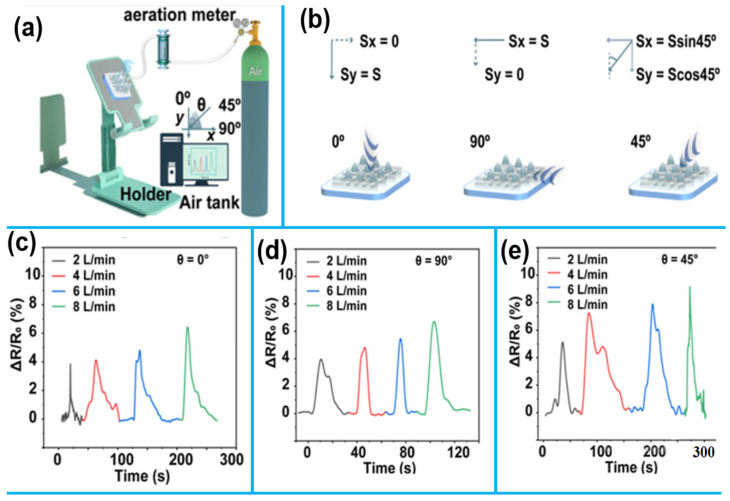
2D Nanomaterials for environmental monitoring using the GSCA to detect airflow. (**a**) The airflow sensing test apparatus’s schematic diagram. The angle between the y-axis and the airflow direction is denoted by θ. (**b**) A schematic examination of the GSCA exposed to various airflow orientations. (**c**–**e**) Changing the angles and aeration rates to 0 (**c**), 90 (**d**), and 45° (**e**), the relative resistance varies with time [100]. Reproduced with permission.

Song et al. [100] used direct ink writing 3D printing to construct a flexible graphene-based sensor with a conical microdot array (GSCA). In addition to improving sensitivity, the 3D structure with microdots enables a quick response time of less than 60 ms. Through the manipulation of printing parameters, the sensor’s sensitivity rose by 32.4% at 26–78 kPa, 800% at 78–102 kPa, and 600% at 102–160 kPa. It also showed excellent linearity between 1.4 and 509 kPa, a broad detection range, and a low detection limit of 11.4 Pa. The spider-leg-like micro-/nanofibers between microdots in the sensor improve electron transport and airflow detection, making it ideal for determining the direction and strength of air in respiration and airflow monitoring. Using an air tank with a flow outlet at different angles, Figure 9a illustrates how the GSCA sensor can measure airflow rates and directions. It accurately detected both airflow strength and direction. In Figure 9b, airflow variations from different directions were broken down into x- and y-axis components. Notably, when the angle θ was set to 0° or 90°, the GSCA predominantly responded to airflow from a single direction. This study demonstrates the potential of conical microdot arrays in graphene-based sensors for airflow detection, respiratory analysis, and real-time personal signal monitoring. MoS_2_, a layered transition-metal dichalcogenide, has also emerged as a promising 2D nanomaterial. Recently, Zhang et al. [114] fabricated self-healable MoS_2_ electrodes using a Schiff base polymer binder. Dynamic imine bonds between polymer chains enabled electrode self-healing through bond breaking and reformation when strained. The electrode fully recovered electrochemical activity within 5 min after being cut. It was successfully applied for persistent heavy metal ion detection in water. Dynamic bonding between graphene/MoS_2_ sheets and functional polymer binders allows electrodes to repeatedly self-repair damage.

#### 1.3.4. 2D Nanomaterials for Energy Storage Application

Batteries and supercapacitors are two examples of energy storage devices that are essential technologies for grid-scale energy storage, electric cars, and portable gadgets. However, capacity fades due to dendrite growth, side products, and mechanical atrophy, which continue as major challenges constraining the cycle lifetime and integrity of these devices [115]. Recently, there has been significant interest in developing self-healing electrodes based on two-dimensional (2D) nanomaterials as a strategy to impart damage tolerance and extend device lifetime. Several distinct self-healing mechanisms enabled by 2D nanomaterials have been reported. Ion or atom diffusion across 2D sheets can repair cracks or defects, restoring electrical conductivity [116].

A self-healing liquid metal-based LIB anode was demonstrated by Wu et al. [117]. An RGO/CNT skeleton supported the Ga-Sn liquid metal alloy that made up this liquid metal. Since Sn reduces Ga’s melting point, the Ga-Sn alloy is self-healing and may be kept as a liquid at 25 °C. During repeated cycling, the RGO/CNT skeleton-enhanced iridium-doped PEDOT with fine-diameter electrodes shows good conductive performance without causing Ga-Sn aggregation or separation. When the Ga-Sn was charged and discharged, it became rougher. Volume expansion explains why Ga-Sn showed a solid state with bumps and ravines during full lithiation. Table 2 below presents an overview of the recent self-healing 2D nanomaterials used for health, motion and environmental applications.

**Table 2:** An overview of recent self-healing 2D Nanomaterials used for health, motion and environmental monitoring.

Materials	Binder	Fabrication Method	Sensitivity	Response Time	Gauge Factor	Strain (%)	Stability (Cycles)	Conductivity	Healing Efficiency	Application	Ref.
rGO	silicone sealant	electrochemical exfoliation	-	395 ms	<40,000	100	1600	-	-	Health	[99]
GSCA	-	3D printing	26–78 kPa	60 ms	-	600	1000	-	-	Health & Environmental	[100]
RGO & (CuNWs)	MF	Coating and in-situ growing	0.088 kPa^−1^	0.3s	-	-	5000	-	-	Health	[101]
rGO	Graphite oxide	touch-sensing mechanism	-	-	-	-	-	28 Ω/square	-	Health	[96]
MXene/PVA hydrogel	Mxene	Gelatinazation	0.40	0.15s	-	200	10,000	-	Excellent	Motion	[102]
GNs	Rubber matrix	metal–ligand coordination	-	-	45,573.1	50	1000	-	Excellent	Motion	[111]
MXene	PU	Spray method	509.8 kPa^−1^	67.3 ms	-	-	10,000		Excellent	Motion	[118]
GO/SnO_2_/PANI	QCM	in-situ oxidative polymerization	29.1 Hz/%RH	7 s/2 s	-	97	-	-	-	Environmental	[119]

QCM = quartz crystal microbalance; CuNWs = copper nanowires; RGO = reduced graphene oxide; MF = melamine foam; GNs = graphene nanosheet; GSCA = graphene-based flexible sensor with a conical microdot array; PU = polyurethane.

### 1.4. Self-Healing Electrodes Based on 3D Nanomaterials

#### 1.4.1. 3D Nanomaterials for Health Monitoring

Three-dimensional (3D) nanomaterials, characterized by their interconnected networks and high surface area, have shown exceptional potential in enabling self-healing properties in electrodes. The unique structural and functional attributes of 3D nanomaterials, such as enhanced mechanical robustness, efficient charge transport, and dynamic interactions at the nanoscale, play a critical role in facilitating self-healing mechanisms. The dimensionality of nanomaterials significantly influences their self-healing capabilities. Unlike zero-dimensional (0D), one-dimensional (1D), or two-dimensional (2D) nanomaterials, 3D nanomaterials possess a porous, interconnected architecture that provides ample space for dynamic molecular interactions, such as hydrogen bonding, π-π stacking, and capillary forces.

**Figure 10:**
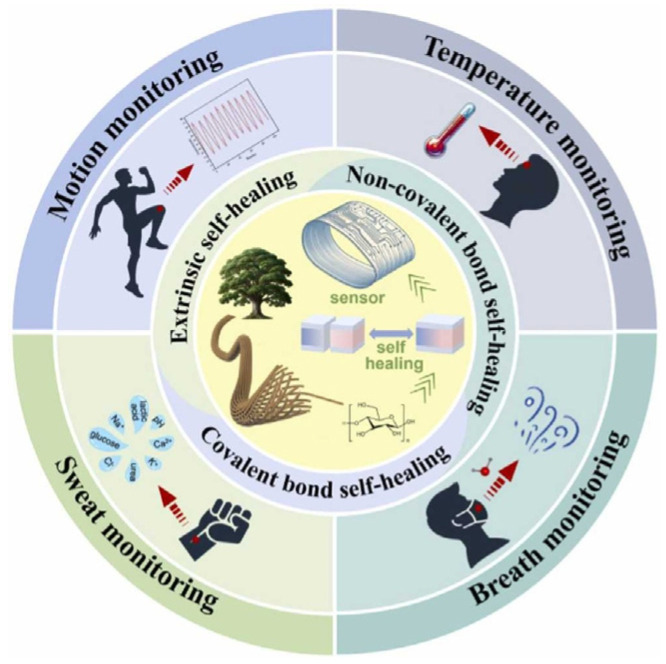
An example of flexible self-healing sensors and how they are used to monitor human health [124]. Reproduced with permission.

Wearable health monitoring devices have become incredibly popular due to their ability to continuously and non-invasively monitor physiological signs. However, the electrodes’ gradual deterioration, which calls for frequent replacement, frequently prevents these devices from being used for extended periods. Research interest in creating self-healing electrodes that regain their functionality following injury has increased as a result [120]. Recent developments in three-dimensional (3D) nanomaterials have demonstrated potential for providing self-healing electrodes. The features of self-healing sensors and their corresponding uses are depicted in Figure 10.

Several recent studies have explored different approaches to impart self-healing capabilities to electrodes. Zhang et al. [121] fabricated a PEDOT: a PSS electrode reinforced with a 3D graphene framework. The electrode could repeatedly self-heal cracks of over 50 μm through capillary forces and π-π stacking interactions between graphene and PEDOT: PSS. In addition to capillary forces, hydrogen bonding has also been utilized for self-healing. In addition to capillary forces, self-healing has also been achieved by hydrogen bonding. AuNPs/MoS_2_/Pep hydrogel, a potent anti-biofouling polypeptide complex hydrogel with exceptional electrochemical properties, was recently developed [122]. It fixes itself completely. Because of these characteristics, the AuNPs/MoS_2_/Pep hydrogel was the best component to use in the electrochemical sweat sensor that prevents fouling [123].

**Figure 11:**
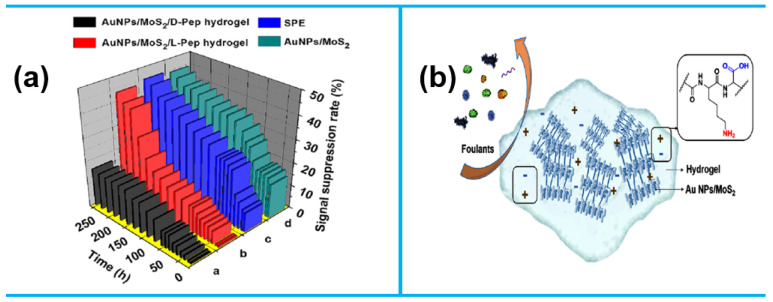
3D Nanomaterials for health monitoring. (**a**) Anti-fouling performance of different electrodes in undiluted human sweat over an extended period. (**b**) Schematic of the designed anti-fouling peptide complex hydrogel [122]. Reproduced with permission.

A recently developed self-healing, anti-biofouling polypeptide complex hydrogel (AuNPs/MoS_2_/Pep hydrogel) offers excellent electrochemical performance [122]. This hydrogel was used to create an anti-fouling electrochemical sweat sensor. The AuNPs/MoS_2_/Pep hydrogel features a network structure (Figure 11a) with numerous hydrophilic groups, making it highly resistant to biofouling in human sweat for up to 30 min and even demonstrates long-term stability in undiluted human sweat (Figure 11b).

#### 1.4.2. 3D Nanomaterials for Motion Monitoring

In 3D nanomaterials like graphene foam, dimensionality significantly enhances self-healing by combining structural and functional advantages that lower-dimensional materials cannot achieve. While damage in 1D nanowires or 2D sheets can completely sever functional pathways, the interconnected 3D network maintains multiple alternative routes for electrical conduction and mechanical load transfer, ensuring partial functionality even during repair. The hierarchical porous structure, with its high surface-to-volume ratio (typically 100–1000 m^2^/g), acts as a reservoir that stores and protects healing agents within its pores until damage occurs. At the same time, the extensive nanoscale interfaces provide enhanced chemical bonding sites, enabling rapid and efficient healing reactions. Furthermore, the 3D architecture serves as a structural scaffold, maintaining geometric integrity during damage and ensuring proper alignment of fractured sections for effective reconnection. These features create an optimal microenvironment for self-healing polymers, encapsulated agents, or vascular networks, achieving healing efficiencies of up to 95% even after multiple damage cycles. This superior performance makes 3D nanomaterials particularly valuable for demanding applications, such as wearable motion sensors, where sustained functionality under mechanical stress is critical.

Novel 3D nanomaterials have been developed for a variety of uses in electronics and sensing, thanks to recent progress in nanotechnology [125]. The application of 3D graphene foam to serve as a scaffold material for self-healing motion sensors has been investigated in several research studies [126]. The highly conductive and porous 3D structure of graphene foam makes it simple to integrate sensing components and self-healing substances.

**Figure 12:**
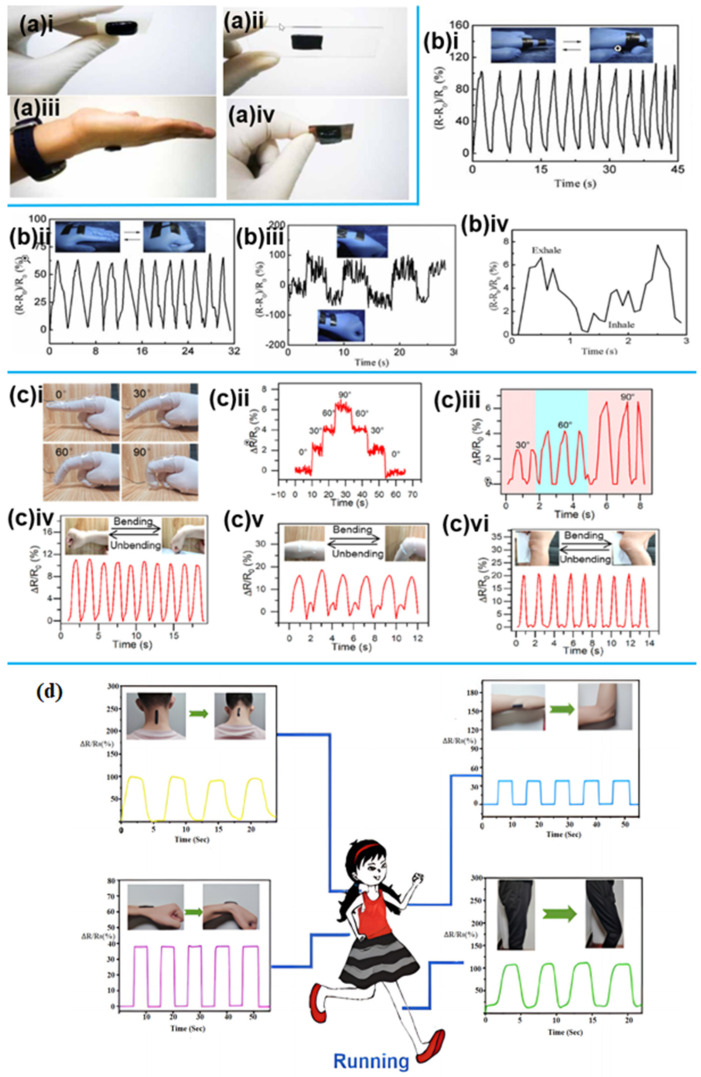
Motion monitoring using 3D nanomaterials. (**a**) PVA-prGO-PDA hydrogels exhibiting the ability to adhere to themselves. The hydrogels adhered steadily to a variety of surfaces. (**i**). Glass; (**ii**). Rubber plate; (**iii**). copper foil; (**iv**). Skins of humans [127]. Reproduced with permission. (**b**) The PLE conductive gels’ real-time resistance ratio for tracking the movements of the (**i**). finger, (**ii**). hand back, (**iii**). wrist, and (**iv**). abdomen [129]. Reproduced with permission. (**c**) (**i**). Images of wireless wearable strain sensors placed at various bending angles on the index finger. (**ii**). The wearable strain sensor reacts resistively to different bending angles. (**iii**). The sensor shows resistive reactions as the fingers bend continuously at various angles. (**iv**). Resistive reactions of the wearable strain sensor to wrist bending. (**v**). Resistive reactions of the wearable strain sensor to elbow bending. (**vi**). Resistive reactions of the wearable strain sensor to knee bending [130]. Reproduced with permission. (**d**) A wearable strain sensor made from PAM@SiO_2_-NH_2_/(ILs-GN) hydrogel for tracking different body movements in real time [131]. Reproduced with permission.

Using conductive rGO, highly elastic PVA, and sticky PDA, Zhang et al. [127] developed a hydrogel-based wearable sensor. With a conductivity of 5 mS cm^−1^, a fracture strain of 2580%, and tensile stress of 146.5 kPa, the hydrogels showed impressive mechanical and electrical properties. Strong and consistent adhesion to a range of surfaces, including human skin, copper foils, glasses, and rubbers, was also demonstrated by the hydrogels (Figure 12a). Moreover, the described flexible sensors exhibit remarkable self-healing capabilities at a rapid pace, independent of the external variables applied. Furthermore, because of their exceptional biocompatibility and self-adhesiveness, these hydrogel-based sensors can detect small-scale movements, such as breathing, swallowing, and pulsing, as well as large-scale motions, such as bending at the knees, knuckles, and necks, without needing to be affixed to human joints and tissues.

In addition to graphene, 3D-printed nanocomposites have also shown promise for self-healing motion sensors [128]. 3D printed nanocomposite containing nickel nanoparticles dispersed throughout a polylactic acid (PLA) matrix. Nickel nanoparticles restored conductivity across cracks developed when the sensor was damaged. The sensor successfully recovered its electromechanical functionality by simulating wound healing. Using a similar methodology, scientists [129] cleverly added conductive PEDOT to create dual physically cross-linked poly(N-hydroxyethyl acrylamide)/Laponite hydrogels: PSS using an on-site doping procedure. This innovative strategy yielded a remarkable multiple physically cross-linked conductive hydrogel system that exhibited an exceptional integration of high-performance characteristics. The resulting PLE conductive gels demonstrated outstanding properties, such as an amazing ripping energy of 1025 J m^−2^, a remarkable extensibility of 23.39, and a quick self-healing speed. Interestingly, after just 6 min at room temperature, the gels were able to restore 82% of their toughness and an astounding 94% of their stiffness thanks to the self-recovery process without the assistance of outside stimuli or interventions. Particularly, 0.5 *w*/*v*% PEDOT: PSS and 5 *w*/*v*% Laponite were used to make these gels. The specimens exhibited an extraordinary capacity for self-healing, as evidenced by their impressive healing efficiency metrics. Remarkably, they demonstrated a 34% healing efficiency in terms of strength recovery, a 54% healing efficiency in strain restoration, and an outstanding 81% healing efficiency in regaining electrical conductivity. Moreover, these exceptional specimens showcased indications of exquisite strain-sensing capabilities, enabling the precise monitoring and observation of intricate human behaviors. From the subtle rhythms of respiration to the intricate articulations of finger and wrist movements, these specimens proved adept at capturing the nuances of human biomechanics, unlocking new frontiers in the realms of wearable technologies, motion tracking, and human–machine interfaces. Given that the PLE conducting gels’ resistance rose with increasing tensile strain, conductive hydrogels can also be utilized to study human motions, including little breaths and wrist and finger movements. Regarding potential applications in electronic wearable sensors, refer to (Figure 12b).

Zheng et al. [130] created self-healing polyampholyte hydrogels for wearable devices, such as capacitive pressure and resistive strain sensors. These hydrogels were synthesized using sodium p-styrene sulfonate (NaSS), (methacryloxyethyl)-trimethylammonium chloride (DMC), and N,N′-methylene bis(acrylamide) (MBAA) as the cross-linker. The resulting NaSS/DMC hydrogels were translucent, highly stretchable, and exhibited both ionic conductivity and self-healing capabilities. These hydrogels were used to create resistive strain sensors with a gauge factor (GF) of 2.9 and excellent cycle stability. They were also utilized to develop capacitive pressure sensors with a GF of 2.17 kPa^−1^ and a detection range of 0–7.35 kPa. By integrating the strain sensors with Bluetooth technology, wearable wireless sensors for tracking human movement were created. This study highlights the versatility of self-healing polyampholyte hydrogels for flexible, high-performance sensors in motion monitoring applications. The resistance was measured using a wireless sensor while the index finger was bent at various angles. The measured resistance values are shown in Figure 12c(i). ΔR/R_0_ increased stepwise when the finger was bent from 0° to 30°, 60°, and 90° and maintained good response stability. A lowering of resistance was caused by a progressive reduction in the bending angle. At a constant bending degree, the ΔR/R_0_ value is nearly constant. This indicates the exceptional reversibility of the strain sensor. There was also evidence of the finger bending cyclically at various angles (Figure 12c(iii)). Similar to this, successful monitoring was also achieved for knee bending (Figure 12c(vi)), elbow bending (Figure 12c(v)), and wrist bending (Figure 12c(iv)).

SiO_2_-NH_2_ spheres and graphene nanosheets (GN) were incorporated into polyacrylamide (PAM) hydrogels by Xie et al. [131] in a related study to produce durable, electrically conductive sensors. The creation of PAM@SiO_2_-NH_2_/(ILs-GN) hydrogels, which showed an exceptional tensile strain of 15,318% and toughness of 51.4 MJ/m^3^, was the consequence of the hydrogen bonding between SiO_2_-NH_2_ and PAM. A rapid reaction to strain was made possible by the sensor’s gauge factor (GF) of 18.94 and strain range of 0.1–1200%. Its conductive system provided excellent sensitivity, enabling it to pick up on minute physiological cues as well as large-scale human movements. The sensor recorded joint movements (neck, elbow, wrist, and knee) in Figure 12d. Resistance variations correlated with deformations, enabling the distinction of joint motions. The sensor’s great sensing capabilities, high strain sensitivity, and outstanding electrical stability highlighted its potential for wearable motion tracking.

While most studies have focused on nanocomposite materials, other approaches have also been investigated in more recent work. For instance, Yu et al. [132] created an ionic liquid-based elastomer composite design to construct a motion sensor that can heal itself. The liquid metal enabled faster reconnection of damaged circuits, while the elastomer offered mechanical strength. This novel design proved effective self-healing with little impact on sensitivity. The ICE-based sensor’s practical performance was tested by attaching it to a puppet for real-time monitoring. As shown in Figure 6a, the sensor detected changes in electronic signals when the puppet’s elbow, knee, and wrist were gently touched and bent. The signals showed repeatability, with the baseline remaining stable during testing. To assess the sensor’s stability under practical conditions, 70 additional experiments were conducted at a consistent 80° wrist bend. The results confirmed that the sensor maintained stable performance throughout, without any significant variations, demonstrating its flexibility, durability, and suitability for long-term use in various applications.

#### 1.4.3. 3D Nanomaterials for Environmental Monitoring

3D nanomaterials are advanced structures with a nanoscale three-dimensional arrangement, characterized by high surface area, porosity, and an interconnected network. These properties make them highly sensitive to environmental changes, enabling effective interactions with pollutants, gases, and toxins through enhanced surface interactions. In environmental monitoring, their porous and flexible structure allows for efficient detection and removal of contaminants, either by absorbing harmful substances or catalyzing reactions to neutralize them. The large surface area ensures rapid response times, even for trace amounts of pollutants, while their 3D architecture supports the development of durable and self-healing sensors. When damaged, these materials can autonomously repair themselves through molecular interactions, such as hydrogen bonding or π-π stacking, ensuring reliable performance in long-term environmental monitoring applications [133].

Environmental monitoring is essential for assessing pollution levels and protecting human and ecosystem health. Electrochemical sensors play a vital role in monitoring various environmental contaminants and parameters. However, these sensors can lose sensitivity and accuracy over time due to electrode fouling and degradation [134]. Recent research has focused on producing self-healing electrodes made of three-dimensional (3D) nanomaterials to provide durable, reusable sensors for continuous environmental monitoring.

**Figure 13:**
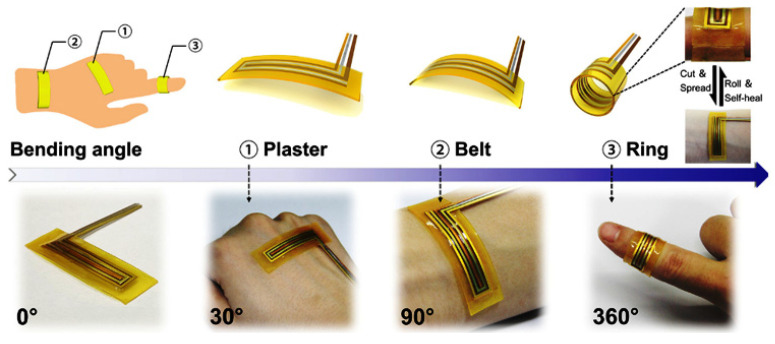
3D Nanomaterials for motion monitoring. Photographs of the convertible detector under different contracting methods [135] Reproduced with permission.

Zhang et al. [135] developed a flexible electrochemical sensor made from ionic polyimine, a recyclable, elastic, and self-healing material, for portable, on-site iodine detection. The sensor’s strong preconcentration ability enables low detection limits and accurate, real-time monitoring of iodine levels. The performance of the sensor is enhanced by the regulation of the ionic polyimine’s cation-anion structure, which is key to its high sensitivity and accurate detection. Additionally, the sensor’s ductility and flexibility make it ideal for integration into a wearable form, facilitating continuous monitoring. An integrated system was developed that includes visual software for real-time data processing and an alarm function for immediate notification in case of abnormal iodine levels, offering practical on-site detection capabilities. It was predicted that a sensing device with this degree of flexibility, repairability, and malleability would make it possible to implant it in wearable electronics and provide real-time exogenous harmful substance detection and alarm. Building on the elastic and flexible characteristics of the IPIN-1 film and electrode materials, a similar three-electrode architecture portable, versatile, and convertible detector was created (Figure 13). A plaster (30°) to a belt (90°) curve was gradually applied to the soft sensor, increasing its bending angle to contact the wrist. Additionally, because of its typical compact circular design and 360° bending degree, the detector may also be utilized as a detecting ring, making it a good choice for use in small places. One noteworthy feature of IPIN-1 film is its rapid self-curing ends, which provide smooth transitions between the ring and other shapes using “cut and spread” or “roll and self-heal” processes.

#### 1.4.4. 3D Nanomaterials for Energy Storage Applications

Three-dimensional (3D) nanomaterials represent a class of advanced materials characterized by a nanoscale architecture that spans all three spatial dimensions, resulting in an interconnected and porous network. This distinctive structure provides them with a high surface area, improved mechanical stability, and effective pathways for charge and mass transport, making them particularly advantageous for energy storage applications, such as batteries and supercapacitors. The three-dimensional nature of these materials is crucial for facilitating their self-healing properties, which enhance their resilience and lifespan.

In contrast to materials with lower dimensions, such as 0D nanoparticles, 1D nanowires, or 2D nanosheets, 3D nanomaterials are equipped with a durable scaffold-like structure that can endure mechanical stress and repetitive cycling without experiencing substantial degradation. This structural robustness is vital for self-healing, as the extensive network provides numerous pathways for both charge transfer and mechanical load distribution, ensuring continued functionality even in the face of localized damage. For instance, in lithium-ion batteries, 3D-nanostructured electrodes can employ dynamic molecular interactions, such as hydrogen bonding, π-π stacking, or reversible covalent bonds, to autonomously repair any cracks or fractures. The high surface area and porous characteristics of 3D nanomaterials create an ideal environment for these healing agents to engage in reactions that effectively restore the material’s functionality.

Energy storage devices are essential technology for a wide range of applications, including consumer electronics and electric vehicles [41]. However, one current obstacle limiting their performance and durability is electrode deterioration due to repeated charge/discharge cycles. Self-healing electrode materials have recently gained popularity as a solution for increasing the cycle life and stability of energy storage devices [136]. Self-healing enables electrodes to repair cracks and defects that occur during repeated electrochemical cycling [137]. Several distinct mechanisms have been utilized to impart self-healing capabilities to electrode materials. One approach relies on dynamic reversible bonding between electrode components. For example, by growing PPy nanoparticles in the GCP framework, after chemically crosslinking the gold nanoparticles with the electrode.

A hydrogel electrode highly responsive to healing polypyrrole-incorporated gold nanoparticle/CNT/poly(acrylamide) (PAM) (GCP@PPy) was developed by Chen et al. [138] and features a hierarchical honeycomb network topology. In addition to the electrode’s exceptional elongation (2380%), Au-SR bonds caused unprecedented visual and electrical healing (94% healing effectiveness). By sandwiching a multifunctional GCP@PPy electrode between a CNT-free GCP (GP) hydrogel electrolyte, a stretchy and real-time omni-healable, an all-gel-state supercapacitor device was developed as a proof-of-concept demonstration.

Furthermore, the researchers included an AgNW film layer that was chemically bonded to the hydrogel electrode. This was accomplished by first spray coating the AgNW film onto the hydrogel, and then using near-infrared (NIR) laser irradiation to firmly attach the current collector layer. Amongst all stretchable supercapacitors that have been studied, this one has the highest energy density (123 Wh cm^−2^) and the largest areal capacitance (885 mF cm^−2^). Beyond the intrinsic elastic and hemostatic characteristics of the electrode and electrolyte materials, the integration of M-SR bonds (where M = Au or Ag) yielded a highly stable and rapidly self-healing structure. This integrated design allowed the device to maintain 89.5% of its initial capacitive performance, even after being subjected to an exceptionally high strain of 800% across ten healing cycles. Remarkably, the supercapacitor prototype was able to recover up to 92% of its initial specific capacitance during the charge-discharge cycling. This demonstrated the first real-time electrical proof-of-concept for an omni-healable supercapacitor device.

One of the most widely studied 3D nanomaterials for energy storage is graphene foam. Its interconnected network provides excellent electrical conductivity and mechanical flexibility, making it an ideal scaffold for self-healing electrodes. For instance, Chen et al. [138] developed a stretchable and self-healing supercapacitor using a 3D graphene framework. The porous structure of the graphene foam allowed for efficient ion diffusion and electron conduction, while its mechanical resilience enabled the device to recover its electrochemical performance after damage. This study demonstrated that 3D graphene structures could withstand repeated mechanical deformation while maintaining high capacitance and energy density, highlighting their potential for flexible and durable energy storage devices.

Similarly, carbon nanotube (CNT) sponges and networks have been explored for their use in self-healing energy storage systems. The high surface area and mechanical flexibility of CNTs make them ideal for creating robust electrodes that can repair cracks and restore conductivity. Huang et al. [91] developed a self-healing supercapacitor using a 3D CNT sponge as the electrode material. The sponge-like structure provided a large surface area for charge storage and enabled the device to recover its performance after mechanical damage. This approach not only improved the durability of the supercapacitor but also demonstrated the potential of 3D CNT networks for creating flexible and high-performance energy storage devices.

In addition to carbon-based materials, 3D metal-organic frameworks (MOFs) have also been investigated for energy storage applications. MOFs are highly porous materials with tunable chemical properties, making them suitable for use in batteries and supercapacitors. Wu et al. [117] developed a self-healing lithium-ion battery anode using a 3D MOF structure. The MOF provided a stable framework for lithium-ion storage and enabled the anode to recover its capacity after mechanical damage. This study highlighted the potential of 3D MOFs for creating durable and high-performance energy storage devices, particularly in applications requiring long-term cycling stability.

Another innovative approach involves the use of 3D nanocomposite hydrogels, which combine polymers with conductive nanomaterials, such as graphene and carbon nanotubes. These materials offer excellent mechanical flexibility and self-healing properties, making them ideal for use in flexible and wearable energy storage devices. Chen et al. [138] developed a self-healing supercapacitor using a 3D nanocomposite hydrogel as the electrode material. The hydrogel contained a network of conductive nanomaterials, which enabled the device to recover its performance after damage. This study demonstrated the potential of 3D nanocomposite hydrogels for creating flexible and durable energy storage devices, particularly in applications requiring high mechanical resilience.

Liquid metals, such as gallium-based alloys, have also been incorporated into 3D nanostructures to create self-healing electrodes for energy storage applications. These materials can flow and reconnect in damaged areas, enabling efficient self-healing. Wu et al. [117] developed a self-healing lithium-ion battery anode using a 3D liquid metal framework. The liquid metal could flow and reconnect at fracture points, restoring the electrode’s conductivity and capacity. This approach not only improved the durability of the battery but also demonstrated the potential of 3D liquid metal structures for creating high-performance energy storage devices.

Emerging developments in 3D-nanostructured electrodes with integrated self-healing functionalities present a viable approach to prolonging the cycle life of battery and supercapacitor devices since many inventive self-healing processes and 3D architectures have been established by current research. Table 3 provides an overview of the recent self-healing 3D nanomaterials used for health, motion, and environmental monitoring.

## 2. Comparative Analysis of Advantages and Limitations of Nano Materials Across Dimensions

Integrating nanomaterials into self-healing electrodes has opened new avenues for advancing sensing and energy storage technologies [148]. The dimensionality of these materials, ranging from zero-dimensional (0D) nanoparticles to three-dimensional (3D) nanostructures, plays a pivotal role in determining their self-healing capabilities, mechanical properties, and functional performance. By examining the unique characteristics of each dimensionality, we can better understand how their structural and functional attributes influence their suitability for specific applications [149].

Zero-dimensional (0D) nanomaterials, such as quantum dots and nanoparticles, are characterized by their high surface-to-volume ratio, which enhances their reactivity and interaction with the surrounding matrix [150]. This property facilitates efficient self-healing mechanisms, as the small size of 0D materials allows them to diffuse rapidly and reconnect at fracture points. However, their lack of structural integrity and tendency to aggregate can compromise the mechanical stability of the electrode. Additionally, while some 0D materials, such as gold nanoparticles, exhibit excellent conductivity, others, such as quantum dots, often require additional functionalization to achieve sufficient electrical performance [151]. Despite these limitations, 0D nanomaterials are widely used in wearable health sensors due to their biocompatibility and optical transparency, as well as in self-healing battery electrodes to repair cracks and restore conductivity [152].

Transitioning to one-dimensional (1D) nanomaterials, such as nanowires and nanotubes, significantly improves mechanical flexibility and electrical conductivity due to their elongated structure. The high aspect ratio of 1D materials enables efficient reconnection at fracture points through mechanisms, such as cold welding or reorientation, making them ideal for flexible strain sensors and environmental monitoring applications [153]. However, integrating 1D nanomaterials into electrodes often requires advanced fabrication techniques, such as layer-by-layer assembly or electrospinning, which can be time-consuming and costly. Furthermore, while 1D materials can reconnect, the healing process may not fully restore the original mechanical or electrical properties, especially after repeated damage. Despite these challenges, 1D nanomaterials, such as motion sensing and electrochemical sensors, are highly effective in applications requiring high stretchability and sensitivity [49].

Two-dimensional (2D) nanomaterials, such as graphene and transition metal dichalcogenides (e.g., MoS_2_), offer exceptional electrical conductivity and mechanical flexibility, making them ideal for flexible electronics. The planar structure of 2D materials provides a large surface area for chemical interactions, enhancing their sensitivity in sensing applications [154]. Additionally, 2D nanosheets can undergo surface reconstruction to repair structural damage, further improving their self-healing capabilities. However, the intrinsic stretchability of 2D materials is often limited, requiring structural modifications, such as buckling or wavy morphologies, to achieve high strain tolerance. Moreover, producing high-quality 2D materials, such as graphene, usually involves complex and costly processes, such as chemical vapor deposition (CVD). Despite these limitations, 2D materials are widely used in wearable devices for continuous physiological monitoring and self-healing supercapacitors and batteries to enhance cycling stability [155].

Finally, three-dimensional (3D) nanomaterials, such as graphene foam and CNT sponges, provide a robust scaffold that maintains structural integrity even after damage, enabling efficient self-healing. The porous structure of 3D materials allows for the storage of healing agents and facilitates rapid ion diffusion, enhancing their performance in energy storage applications [156]. The 3D architecture also distributes mechanical stress more evenly, reducing the risk of catastrophic failure and improving the durability of self-healing electrodes. However, synthesizing 3D nanomaterials often involves intricate processes, such as templating or 3D printing, which can be expensive and time-consuming. Additionally, many 3D self-healing systems rely on extrinsic healing agents, which may be depleted over time, limiting their long-term effectiveness. Despite these challenges, 3D nanomaterials, such as motion sensors and energy storage devices, are highly effective in applications requiring mechanical resilience and high porosity [157].

However, the choice of nanomaterial dimensionality depends on the application’s specific requirements. While 0D and 1D nanomaterials offer simplicity and ease of integration, 2D and 3D materials provide superior mechanical and electrochemical properties. Future research should focus on overcoming the limitations of each dimensionality, such as improving the scalability of 3D nanomaterials and enhancing the stretchability of 2D materials. By utilizing the distinct benefits of each dimensionality, the next generation of self-healing electrodes can attain previously unheard-of performance in energy storage and sensing applications [158]. Table 4 below compares the self-healing mechanism, fabrication methods, and self-healing performance of different OD,1D, 2D, and 3D nanomaterials for various applications.

## 3. Balancing Mechanical Robustness and Self-Healing Kinetics in Advanced Polymeric Materials

Self-healing materials have emerged as a transformative solution for extending the durability and sustainability of polymeric materials. However, a fundamental challenge persists in balancing mechanical robustness with efficient self-healing kinetics. Highly crosslinked polymer networks provide excellent mechanical strength but restrict molecular mobility, impeding self-repair efficiency. Conversely, materials designed for rapid self-healing often lack the structural integrity required for practical applications [163].

The self-healing capability of polymeric materials depends on the movement and diffusion of polymer chains, as well as the dissociation and recombination of dynamic bonds. In highly crosslinked networks, strong covalent bonds enhance mechanical strength but limit the reconfigurability needed for self-healing. Li et al. emphasize that intrinsic self-healing mechanisms must be carefully engineered to strike an optimal balance between strength and repairability [164].

Intrinsic self-healing materials rely on reversible covalent bonds (e.g., Diels-Alder reactions, disulfide bonds) and non-covalent interactions (e.g., hydrogen bonding, metal coordination) to facilitate repeated self-repair. However, stronger crosslinking density reduces segmental mobility, leading to slower self-healing kinetics. To address this, Li et al. suggest adopting hybrid strategies that integrate dynamic bonds within hierarchically ordered structures inspired by biological tissues.

### Biomimetic Approaches for Balancing Strength and Self-Healing

Natural systems, such as cartilage and tendons, exhibit remarkable mechanical properties while retaining self-healing abilities due to their hierarchical architectures and dynamic bonding networks. Li et al. (2024) [164] highlight several key biomimetic strategies:

i. 3D Interconnected Network Structures: Inspired by cartilage tissue, which integrates rigid collagen fibrils with flexible proteoglycans, synthetic polymeric materials can incorporate interwoven networks of nanofillers to maintain mechanical strength while facilitating repair. The study discusses the use of tungsten disulfide (WS_2_) nanosheets embedded in a polyurethane matrix, forming a hydrogen bond-driven 3D skeleton that enhances both durability and healing efficiency.
ii. Hybrid Crosslinking Strategies: Combining permanent covalent crosslinks with reversible dynamic bonds allows selective breakage and reformation of specific bonds, ensuring structural stability while enabling molecular rearrangement. For example, the integration of supramolecular interactions (e.g., host-guest chemistry) within rigid frameworks improves resilience without sacrificing healability.
iii. Gradient and Multiscale Structural Engineering: Inspired by fish scales and nacre, gradient architectures provide a balance between rigidity and flexibility by varying crosslinking densities across different layers. Li et al. [164] explore the role of anisotropic structural hierarchies in distributing stress efficiently while maintaining repair potential.
iv. Dynamic Interfacial Bonding: Strengthening interfacial interactions between polymer chains and nanofillers reduces the risk of mechanical failure while enhancing molecular diffusion during healing. The use of tannic acid-modified nanofillers to improve hydrogen bonding interactions exemplifies a practical approach discussed in the study.

## 4. Conclusions and Perspective

Nanomaterial-based self-healing electrodes have given new impetus to sensing and energy storage domains by presenting solutions to major problems, namely, mechanical stress, material aging, and device lifetime. The unique properties of nanomaterials, including their high surface area, electrical conductivity, and mechanical flexibility, have enabled the development of advanced electrodes capable of recovering their functionality after damage. These electrodes also contain several dimensional nanomaterials, including zero-dimensional nanoparticles (e.g., quantum dots, gold nanoparticles), one-dimensional nanowires (e.g., silver and copper nanowires), two-dimensional nanosheets (e.g., graphene, MoS_2_) and three-dimensional nanostructures (e.g., CNT sponges and graphene foams). All these materials contribute unique properties to self-healing systems, such as improved conductivity, flexibility, and kinetic energy storage mechanisms (hydrogen bonding, ionic interactions, and dynamic covalent bonds). Combining polymers with chemically functionalized bonds and liquid metals tailors the versatility and performance of these self-healing systems to also robustly address real-world applications.

Evidence of the effect of self-healing electrodes has been seen in a wide variety of applications. In health monitoring, for example, self-repairing bioelectrodes integrated into wearable devices allow continuous monitoring of physiological measurements, e.g., ECG/EMG, without degrading performance even under repeated deformation. In motion sensing, for example, the same issues arise when flexible and stretchable sensors enable strong detection of movements under high mechanical stress, which emphasizes their multidisciplinary importance in the biomedical and industrial domains. Environmental sensors also gain from robust self-healing sensors, which increase the reliability and lifespan of devices for monitoring environmental pollutants. At the same time, in energy storage, batteries and supercapacitors with self-healing electrodes exhibit improved cycling stability, which efficiently solves the problems of capacity depletion and mechanical damage. These developments point to the revolutionary ability of self-healing electrodes to bring about more robust, efficacious, and environmentally friendly technologies.

Despite these significant achievements, challenges persist in realizing the full potential of self-healing electrodes. Scalability continues to be a significant barrier since cost-effective and scalable fabrication is crucial for commercialization. Long-term stability and biocompatibility of such materials, in particular for wearables and biomedical use, are also an area in need of research and development. Additionally, the seamless fabrication of self-healing materials onto conventional devices and/or systems is crucial in real-world applications. Performance optimization, such as electrical, thermal, and mechanical properties where improvements need to be equal to or better than those of conventional materials, continues to be another focus.

Looking to the future, the development of advanced materials with improved self-healing efficiency, conductivity, and environmental adaptability will be a key focus. Designs that can perform in real time and be used for damage detection and self-repair, as well as for adaptive performance optimization are likely to become the next generation. Hybrid systems comprising multiple nanomaterials and self-healing functionalities represent an exciting avenue for functional and structural versatility and high performance. In addition, the adaptation of self-healing electrode designs, when needed, to particular application demands (e.g., flexible electronics, energy harvesting, bio-implants) will stimulate innovation and extend their scope of applications.

Owing to continuous improvements in material science and engineering, self-healing electrodes made of nanomaterials are well positioned to make significant contributions to the development of sensing and energy storage technology in the future. Through improvements upon device reliability, life-span, and enabling eco-friendly solutions, these electrodes are moving from a research idea to a reality for a sizable impact. The collaborative efforts of researchers and engineers worldwide will ensure that self-healing electrodes become an integral part of next-generation technologies, transforming our approach to device durability and performance.

## Figures and Tables

**Table 3:** An overview of recent self-healing 3D Nanomaterials used for health, motion and environmental monitoring.

Material	Fabrication	Conductivity	Healing Efficiency (%)	Response Time	Tensile Strength	Gauge Factor	Strain (%)	Application	Ref.
PD embedded hydrogel	Copolymerizat-ion	6.8 mS cm^−1^	2.32	**-**	**-**	1.09	585	Health	[25]
AuNPs/MoS_2_/Pep hydrogel	**-**	-	-	100 s	**-**	-	55	Health	[122]
Ionogel	polymerization	0.21 mS/m	98	200 ms	**-**	1.05	0–400	Health	[139]
Nanocomposite of Ag–Au nanowires and SBS elastomer	ligand exchange	41,850 S cm^−1^	-	-	**-**	-	266	Health	[140]
graphene nanocomposite hydrogels	facile two-step	5 mS cm^−1^	98	10 s	146.5 KPa	-	2580	Motion & Health	[127]
nanocomposite hydrogels	in situ doping	0.04–0.09 S m^−1^	81	-	34	-	54	Motion	[129]
Polyampholyte Hydrogels	free-radical polymerization	0.015 S cm^−1^	-	250 ms	0–7.35 kPa	2.9	0–350	Motion	[130]
IL-based conductive elastomers	one-pot Pickering emulsion polymerization.	-	98	200 ms	-	1.05	0–400	Motion	[132]
Ionic hydrogel	One-pot	-	96	2 h	-	9.0	2100	Motion	[141]
TOCNF/PAA-PPy composite hydrogel	facile combined two-step preparation	3.9 S m^−1^	99.4	6 h	0.55 MPa	7.3	889	Motion	[142]
MXene-boosted PAA hydrogel	Swift assembly	-	80	1.5 s	-	1.08	500	Motion	[143]
zwitterionic hydrogel	one-step immersion	0.16–1.65 S m^−1^	30–70	-	3.2–202 kPa	9.1	1000–2880	Motion	[144]
Dual Noncovalent Network Elastomer	-	-	80	30 s	-	-	0.2	Motion	[145]
multifunctional conductive hydrogels	Uniform dispersion	-	94	15 s	-	-	>1000	Motion	[146]
PAAm hydrogel	-	59.55 mS·cm^−1^	99	150 ms	-	6.44	>900	Motion	[147]
PAM@SiO2-NH2/(ILs-GN) hydrogel	One-pot	12 mS/cm	75.01	-	1057 KPa	18.94	1200	Motion	[131]
IPIN	three-electrode system	1.98 × 10^−2^ mS cm^−1^–4.91 × 10^−3^ mS cm^−1^	-	8s	4.28 mPA	-	0–250	Environmental	[135]

IPIN = ionic polyimine network; IL = Ionic liquid; PAAm = poly(acrylamide).

**Table 4:** Comparison of self-healing mechanism, fabrication methods, and self-healing performance of different OD,1D, 2D and 3D nanomaterials for various applications.

Electrode	Healing Mechanism	Fabrication Methods	Damage/Healing Method	Healing Efficiency	Healing Time	Stability	Application	Ref.
OD &1D Nanomaterials								
cPDA@ZPD NPs	dynamic hydrogen bonding interactions	copolymerization	Cut/healing	2.32%	-	12,000	Health monitoring	[25]
MWCNT	π-π stacking interactions	Laser Ablation	Crack Healing	90% Conductivity Recovery	<5 min	5000 cycles	Flexible Electronics	[159]
FHE nanofiber	hydrogen bonding and electrostatic interactions	Electrospinning	Cut/Scratch/healing	98.3%	-	3000	Health Monitoring	[60]
AgNPs	Metallic bonding interactions	Screen Printing	Cut/Healing	85% Conductivity Recovery	10 min	8000 cycles	Motion Monitoring	[36]
AuNWs	Surface diffusion healing	Deposition on Latex Rubber	Crack Healing	95% Resistance Recovery	2 min	10,000 cycles	Wearable Biomedical Sensors	[58]
IrNPs	Metallic bonding interactions	Atomic layer deposition	-	-	150 ms	>10,000	Motion Monitoring	[66]
CNT/Polymer Composite	Dynamic covalent bonding	Solution Processing	Cut/Healing	92% Conductivity Recovery	30 min	15,000 cycles	Wearable sensors	[160]
2D Nanomaterials								
RGO	π-π stacking and hydrogen bonding	Electrospinning	Crack Healing	87% Conductivity Recovery	5 min	10,000 cycles	Health Monitoring	[60]
GO	Hydrogen bonding interactions	Solution Processing	Crack Healing	80% Conductivity Recovery	15 min	8000 cycles	Wearable Electronics	[78]
Mxene	Surface functionalization repair	Vacuum-Assisted Filtration	Cut/Healing	94% Conductivity Recovery	8 min	12,000 cycles	Flexible Sensors	[161]
GNs	π-π interaction	Chemical Vapor Deposition	Crack Healing	96% Conductivity Recovery	2 min	15,000 cycles	Wearable Electronics	[68]
MoS_2_	Van der Waals interactions	Solution Processing	Cut/Healing	89% Conductivity Recovery	6 min	10 cycles	Flexible Sensors	[162]
3D Nanomaterials								
Hydrogel	Hydrogen bonding interactions	Copolymerization	Cut/Healing	4.9× Conductivity Increase	-	12,000 cycles	Health Monitoring	[25]
Ionogel	Ionic crosslinking	Polymerization	Crack Healing	92% Conductivity Recovery	5 min	10,000 cycles	Wearable Strain Sensors	[139]
IL-based conductive elastomers	Reversible ion interactions	Solution Processing	Crack healing	88% Conductivity Recovery	7 min	8000 cycles	Stretchable Electronics	[132]
